# The UF/MSK adult mesh-based computational human phantom library: applications to organ dosimetry in computed tomography

**DOI:** 10.1088/1361-6560/ae1dc5

**Published:** 2025-11-21

**Authors:** Robert J Dawson, Jared M Baggett, Laura E Dinwiddie, Wyatt W Smither, Yitian Wang, Stefan K Wehmeier, Trung Tran, Shreya Pathak, Sean J Domal, Cameron Kofler, Chansoo Choi, Yeon Soo Yeom, Lukas M Carter, Juan C Ocampo Ramos, Pat B Zanzonico, Adam L Kesner, Wesley E Bolch

**Affiliations:** 1 Medical Physics Program College of Medicine, University of Florida, Gainesville, FL, United States of America; 2 J. Crayton Pruitt Family Department of Biomedical Engineering, University of Florida, Gainesville, FL, United States of America; 3 Department of Radiology and Imaging Sciences, Indiana University, Indianapolis, IN, United States of America; 4 Department of Radiation Oncology, University of Texas Southwestern Medical Center, Dallas, TX, United States of America; 5 Department of Radiology, University of Chicago, Chicago, IL, United States of America; 6 Department of Radiation Convergence Engineering, Yonsei University, Wonju, Republic of Korea; 7 Department of Medical Physics, Memorial Sloan Kettering Cancer Center, New York, NY, United States of America

**Keywords:** computed tomography, organ dosimetry, dose coefficients, computational phantoms

## Abstract

**Objective.:**

To develop a comprehensive mesh-based computational human phantom library of adult males and females that span current US body-size distributions. The phantom library was developed jointly between the University of Florida and Memorial Sloan-Kettering Cancer Center and termed the UF/MSK Phantom Library.

**Approach.:**

Adult phantoms were modeled by rescaling and systematically deforming the International Commission on Radiological Protection Publication 145 mesh-type reference computational phantoms to match heights, body mass, and CDC National Health and Nutrition Examination Survey-derived secondary anthropometric parameters. Low body-mass phantoms underwent uniform two-dimensional scaling of abdominal organs to avoid collision with subcutaneous fat. To accommodate larger body circumferences, a rigging procedure was developed to smoothly deform the limbs while maintaining anatomical realism and preserving tissue. A companion ‘arms-up’ version of the library appropriate for computed tomography (CT) dosimetry simulation was also constructed. A validated CT source term was developed for the Canon Aquilion ONE/GENESIS considering technique factors such as tube voltage, bowtie filter size, and beam collimation, and was used to compute slice-specific organ doses for a subset of library phantoms.

**Main results.:**

A large library of 186 male and 171 female (357 total) phantoms was created. Standing height ranged from 150–200 cm for males and 150–195 cm for females, and total body mass ranged from 40–150 kg for males and 40–155 kg for females. Simulated CT organ doses varied smoothly with BMI for a fixed phantom height.

**Significance.:**

The UF/MSK adult phantom library has been constructed with body morphometries representative of the US population. Organ doses computed for phantoms of varying body sizes demonstrate the importance having a morphometrically robust selection of computational phantoms for patient matching. While the immediate application of this library is to expand phantom selection options in MIRDct software (www.mirdsoft.org), the library can be further utilized in other applications including radiopharmaceutical, radiography, fluoroscopy, and radiotherapy organ dose reconstructions.

## 1. Introduction

Computational human phantoms have evolved substantially over the past decades, beginning with stylized, surface equation-based models which were later superseded by voxelized models derived from patient medical computed tomography (CT) or magnetic resonance images (Xu 2014). Recently, the International Commission on Radiological Protection (ICRP) published adult male and female mesh-type reference computational phantoms (MRCPs) for use in radiation dosimetry studies (ICRP 2020). These were later expanded by the ICRP to include a fully mesh-based series of ICRP reference pediatric phantoms (ICRP 2024). The mesh format confers several advantages over voxel predecessors: it enables explicit modeling of sub-millimeter radiosensitive layers and controlled deformations/posture changes of tissue surfaces. It also avoids grid-induced stair-step artifacts that can bias transport at curved boundaries. For example, when cortical bone thickness approaches the voxel size, partial-volume effects can under-represent the cortical layer and its shielding of the underlying red marrow, biasing active-marrow dose upward for external photon irradiation. Tetrahedral mesh phantoms have also been shown to provide superior computational speed compared to voxelized models in the context of Monte Carlo radiation transport simulations (Yeom et al 2014).

While phantom libraries have been developed using stylized or voxel-based technologies to represent various human populations, there are currently very few computational human phantom libraries in fully mesh-type formats. Examples of polygon mesh-type phantom libraries of adults and/or children include those presented by Johnson et al (2009), Na et al (2010), Cassola et al (2011), Segars et al (2013), Geyer et al (2014), Segars et al (2015), Chen et al (2016), Xie et al (2017), and Dahal et al (2025). At present, there are three phantom libraries specifically given in tetrahedral mesh-type formats, and these include the computational phantom series of Lee et al (2019), Choi et al (2020), and most recently Kim et al (2024).

CT is indispensable in modern medicine, yet its ionizing radiation motivates continued dose optimization and transparent risk communication. Dose-reduction initiatives such as Image Gently and Image Wisely reflect these concerns (Goske et al 2008). Patient dose varies strongly with body size, and widely adopted size surrogates and metrics—such as size-specific dose estimate and water-equivalent diameter—capture much of this dependence (Boone et al 2011, Christner et al 2012, McCollough et al 2014). A population-spanning, deformable, mesh-type phantom library that reproduces realistic secondary anthropometrics (torso and limb circumferences, sitting height) is therefore a practical foundation for scanner- and protocol-specific organ-dose estimation, including common clinical postures (arms-down and arms-up) used in CT.

In the present study, we introduce the University of Florida/Memorial Sloan-Kettering (UF/MSK) male and female adult tetrahedral phantom libraries, which expand upon the adult UF/NCI phantom library of Geyer et al (2014), both in terms of the number of phantoms and granularity of their individualized standing heights and total body masses. The adult members of the earlier UF/NCI hybrid library were non-tetrahedral NURBS/polygon phantoms constructed from different starting anatomies. By contrast, the UF/MSK library begins from the ICRP 145 MRCPs and applies National Health and Nutrition Examination Survey (NHANES)-guided morphing in fully mesh-type format; the two libraries therefore differ in origin and mesh representation while sharing the goal of population coverage.

This new phantom library targets the body size distribution of the current United States adult population, and is based upon morphometric rescaling of the ICRP Publication 145 adult male and female reference phantoms (ICRP 2020). To take full advantage of the novel deformability of mesh phantoms, a workflow was developed to rotate the limbs of the phantoms in an anatomically realistic fashion, allowing for the construction of a one-to-one companion library comprised of the same phantoms in an arms-raised posture as is common in clinical CT examinations.

To demonstrate the utility of the UF/MSK phantom library in the context of CT dosimetry, a scanner-specific Monte Carlo source term was first developed and validated for a Canon Aquilion ONE GENESIS system using physical measurements of beam output. Monte Carlo radiation transport simulations were performed to compute slice-specific organ absorbed doses for a subset of phantoms from the library, and total organ doses from several commonly used clinical protocols were computed by accumulating slice-specific doses over the corresponding scan ranges. This study further demonstrates the application of such a diverse phantom library in deriving CT organ dose estimates by comparing body mass-parameterized doses to those produced using a single adult reference individual. Among other applications, more accurate CT absorbed-dose estimates achievable using tetrahedral mesh-type format phantom can contribute to clarifying this important issue.

## 2. Methods

### 2.1. Derivation of primary and secondary scaling targets from CDC NHANES data

To develop a library of computational mesh phantoms that morphometrically represent the current US adult populations, anthropometric data were obtained from the NHANES^8^. NHANES contains health and demographic data from a representative population of individuals, including body morphometry data, which define morphometric targets in the UF/MSK adult phantom library. The library is divided into two sub-populations: adult females and adult males. After parameterizing the NHANES anthropometric data for each sub-population, the ICRP Publication 145 mesh-type reference phantoms were computationally morphed to fulfill morphometric targets.

Anthropometric data were acquired from the NHANES III series with 31 311 participants acquired during the years 1988–1994 and from the continuous NHANES series with 88 062 participants acquired during the years 1999–2016. Participants of age 19 and older were grouped within the adult sub-populations. Table 1 indicates the anthropometric parameters that the current work adopted from each NHANES series, where the continuous NHANES was delineated in two-year increments. Some notable NHANES parameters that were not adopted are the following to include (1) body mass index, which is derived from standing height and body mass, (2) recumbent length, which applies to infants only, and (3) head circumference, which is only available between the ages of 2–7 years.

Participant data were sorted into the two sub-populations. For a given sub-population, a computational mesh phantom was identified by its standing height and total body mass, which were termed primary parameters. Such phantoms were then made to fulfill the remaining parameters from table 1, termed secondary parameters. Additional parameters not yet mentioned, such as forearm circumference, chest circumference, and neck circumference, were considered free parameters. The objective was to parameterize each secondary parameter in terms of one or both primary parameters for a given sub-population. Once a parametric fitting equation was defined, a given phantom’s identifying primary parameters were substituted into the regression equation to evaluate their corresponding secondary parameters.

The ratio of mass to standing height is more closely associated with cross-sectional area than with circumference metrics, suggesting that a nonlinear (e.g. square-root or low-order polynomial) relationship should be expected between the primary and secondary parameters. Despite this, a linear regression approach was chosen because it was supported empirically, as shown in table 2, while attempted fits to nonlinear functions did not provide significant or consistent gains in correlations. While the dominant dosimetric driver in the context of CT irradiation is cross-sectional attenuation, this relationship was captured by the selected circumference metrics. The linear mapping was sufficiently accurate and avoided overfitting while providing smooth, monotonic targets for later morphing of the phantoms’ outer body contours.

For each sub-population’s secondary parameters, a linear regression was applied to fit the secondary parameter to one primary parameter or a function of both primary parameters. The general approach was to fit length parameters to standing height and to fit circumferential parameters to the ratio of total body mass to standing height. Table 2 lists the slope and ordinate intercept for linear equations of fitted secondary parameters. A summary of the final primary and secondary anthropometric parameters can be found in Annex A (males) and Annex B (females). Secondary parameters modeled as functions of primary parameters, with coefficients of determination greater than 0.5, were selected as potential modeling parameters. Ultimately, sitting height and circumferences of the calf, thigh, upper arm, and buttocks were selected to define the phantoms’ anthropometric parameters.

With secondary parameters modeled in terms of primary parameters, the next step was to determine the primary parameters that identify each phantom in the library. Primary parameters were defined in increments of 5 cm for standing height and 5 kg for total body mass. For each sub-population, the 5th and 95th percentiles of standing height were calculated, and the standing height bins were defined by rounding these percentiles to the nearest multiple of 5 cm, as shown in table 3. Similarly, the total body mass bins were calculated in increments of 5 kg to include the 5th and 95th percentile of total body mass for each standing height bin, as shown in table 4 and in table 5. This concluded the initial selection of target phantoms for the new mesh-phantom library. Following this set of target standing height and total body mass combinations, the library was further expanded to include more extreme primary scaling targets based on participants in the National Lung Screening Trial, for which the UF/NCI adult phantom library was deemed to be insufficient (not covering the full range of body sizes of the study cohort) (Lee et al 2017).

While organ dose generally depends on cross-sectional attenuation rather than height or body mass directly, previous studies and AAPM task group reports have shown that simple size surrogates—effective diameter *D*_eff_ or water-equivalent diameter *D*_w_ —capture the principal variation in dose across patients and scanners (Boone et al 2011, Christner et al 2012, McCollough et al 2014). The described workflow uses height and body mass as primary parameters because they are ubiquitous in clinical records and, via the fitted secondary anthropometrics (table 2), reproduce population-averaged distributions in torso circumferences and indirectly AP/LAT thickness that determine *D*_eff_ or *D*_w_. Thus, library selection by height/body mass is a practical proxy for selection by size-dependent attenuation.

### 2.2. Phantom modeling

**Figure 1.**
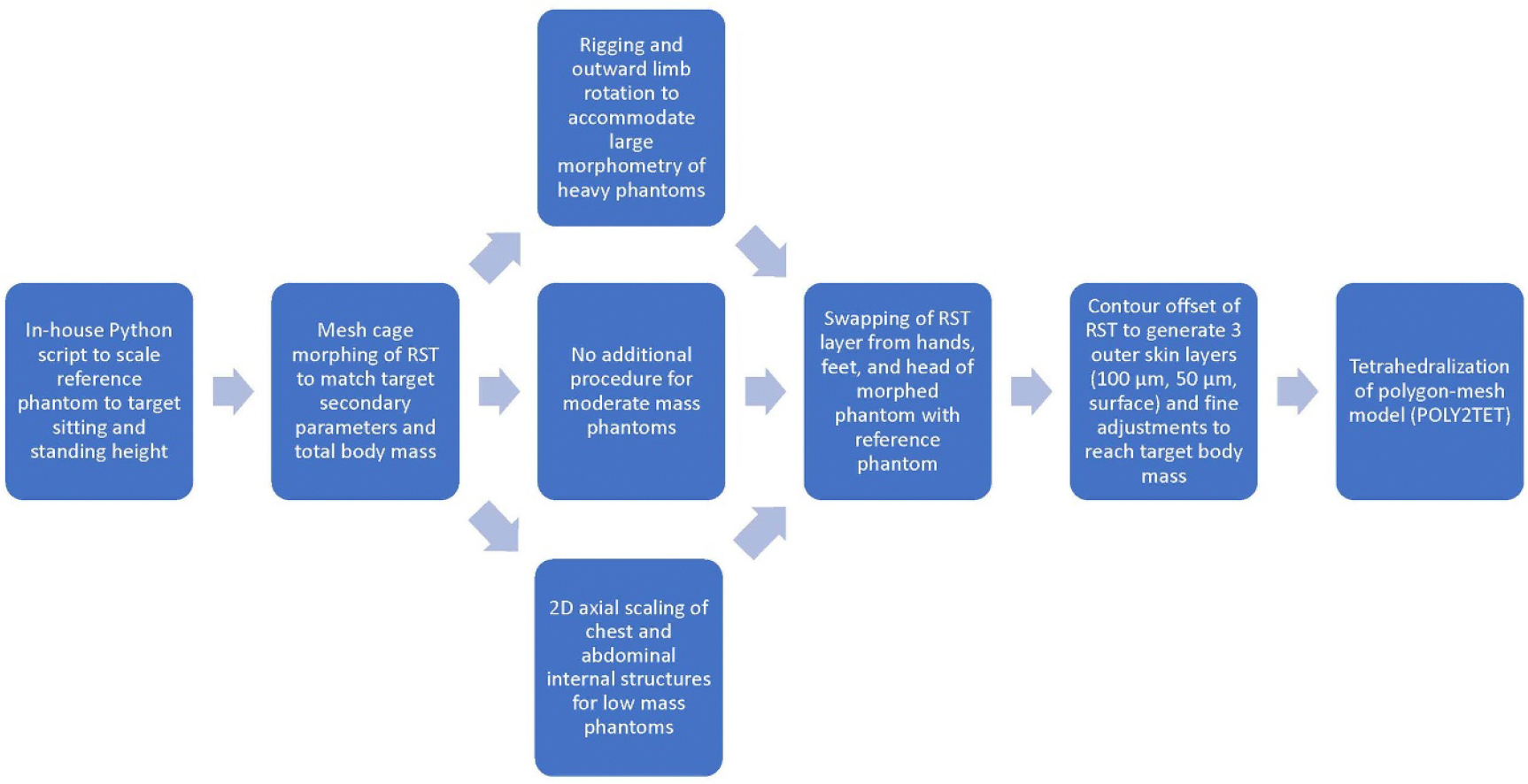
Workflow of the phantom development process.

Beginning with the ICRP Publication 145 adult male and female MRCPs, mesh phantoms of the UF/MSK library were created using a series of automated, semiautomated, and manual morphing processes. A flowchart of the modeling process is shown in figure 1. For a given phantom in the UF/MSK library, the target sitting height was matched by first uniformly scaling the base MRCP in three dimensions (*x, y*, and *z* directions) by a scale factor defined as the ratio of target and initial sitting heights. The bilateral ischial centroid (the midpoint of the base of the pelvis) served as the geometric origin for this scaling. The lower body of the resulting phantom was then one-dimensionally scaled in the *z*-direction to match overall standing height. All uniform scaling of the adult MRCPs was performed using in-house Python scripts that directly modified the positions of vertices (and thus facets) in the polygon meshes.

Once a given phantom had been scaled to match the targeted sitting and standing heights, a hyper-simplified mesh model (with a number of vertices on the order of 100) was constructed and aligned with the bony anatomy of the phantom. Using a process known as ‘mesh cage morphing’ in the software code Rhinoceros 3D (www.rhino3d.com) and its associated visual programming language Grasshopper. Vertices of the residual soft tissue (RST)^9^ layer, representing the outermost layer of subcutaneous adipose tissue (SAT), were mathematically associated with nearby vertices of the mesh cage, such that controlled deformations of the cage led to concomitant deformations in the RST mesh. The RST layer, which closely underlies the outermost skin layer the phantoms, represented the outer body contour of the virtual individual. By monitoring the circumferences of various body parts in the RST, the layer was able to be recontoured to match the secondary anthropometric parameters prescribed for that standing height and total body mass combination in real time. Limb circumferences, such as those for the upper arm and thigh, were defined at the midpoint of the associated long bone (e.g. the femur for thigh circumference). Waist circumference was defined at the most superior z-extent of the pelvis, while calf circumference was defined at the most superior z-extent of the tibia’s medullary cavity.

A complicating side effect of the mesh cage morphing procedure was that RST vertices within the hands, feet, and head of the phantom were slightly deformed, causing intersections with the closely underlying muscle mesh. To resolve these issues, the same structures in the phantom prior to mesh cage morphing were isolated using a Boolean subtraction function in Rhinoceros 3D and excluded from this deformation process. These unmodified regions were then swapped with the deformed structures in the phantom following the mesh cage morphing step. Next, the outer three skin mesh layers (exterior surface, 50 *μ*m deep dead cell basal interface, and the 100 *μ*m deep basal cell to dermis interface) were generated using a mesh offset command in either the Rhinoceros 3D or Blender 3D software (www.blender.org). Any intersections in the resulting phantom, which most commonly occurred in high-curvature areas such as the groin and axillae, were then located and manually repaired prior to tetrahedralization. Final adjustments to the RST and skin layers were then made (preserving the thickness of each layer) to match the phantom’s overall total body mass to within ±1% of the target mass by iteratively tetrahedralization of the model and re-calculating the total body mass. Tetrahedralization was performed using the software code POLY2TET (Han et al 2020).

**Figure 2.**
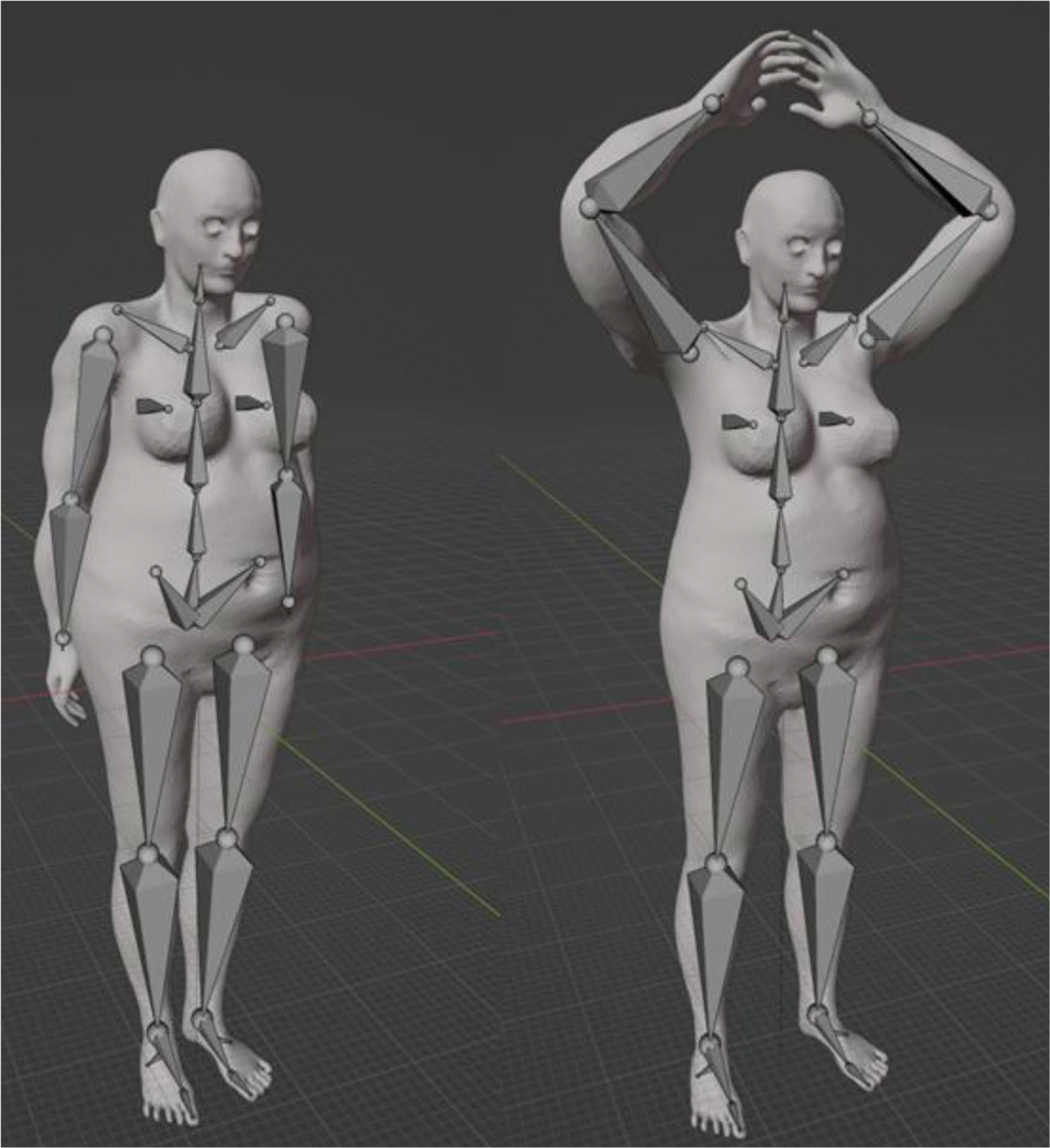
Example of the anthropometric rig used for anatomically realistic limb deformation in Blender 3D. ‘Solid rigging’ (absolute transformation with the rig) was employed to transform bone tissues, while smooth gradient weights were assigned to soft tissues to include the muscle, skin, RST, and blood vasculature.

For moderate-to-high total body mass phantoms, a problem was encountered wherein the expanding chest and abdominal regions began to intersect with structures in the arms. Since any mesh intersections prevent the model from being tetrahedralized, an anthropometric mesh rigging procedure was developed in Blender 3D to realistically deform the limbs of the phantoms outward to prevent such intersections. For each MRCP, a mesh rig was constructed and its points of rotation (in the areas of the shoulder, hip, and elbow) manually aligned carefully with the anatomical joints in the phantom. An example of a typical anthropometric rig used is shown in figure 2. Depending on their position relative to the associated rig segment (termed ‘bone’ in Blender 3D), vertices in the phantom’s meshes were given a weight between 0 and 1 inclusive, such that the former signifies no transformation with the ‘bone’, the latter absolute transformation with the ‘bone’, and any weight between 0 and 1 a partial transformation with the ‘bone’. In this manner, smooth and anatomically realistic mesh deformations around the points of rotation were achieved without inducing mesh-mesh or self-intersections. Skeleton (cortical, spongiosa, and medullary cavity) meshes had 1 assigned for all vertex weights to ensure complete preservation of mass and shape, while muscle, skin, and vasculature meshes were weighted with smooth gradients between 0 and 1 using the ‘automatic vertex weighting’ function in Blender 3D, which is visually realistic for humanoid meshes.

As meshes must be closed manifolds (with consistent, outward facing vertex normals) in order to be valid and tetrahedralizable, the muscle mesh in the MRCPs has vertices surrounding all objects that penetrate its surface at a 50 *μ*m offset. For example, at sites where the blood vasculature penetrates the muscle surface, the muscle mesh surface enters the bulk mesh and ‘lines’ the vasculature. Since the automatic vertex weighting cannot handle these complex geometries while preserving the muscle offset, a primitive version of the muscle meshes was provided by Hanyang University, which lack vertices surrounding soft tissues internal to the muscle surface (facilitating both blood vasculature and lymph nodes). The primitive muscle meshes were further modified to lack vertices surrounding cortical bones in the arms and shoulders that were transformed during the rigging process. Once all of the meshes were positioned appropriately, an offset of the aforementioned excluded meshes was performed and then Boolean subtracted from the muscle mesh to yield a final muscle model. Meshes that were deformable rigged (muscle, vasculature, RST, and skin layers) were manually adjusted such that their final volume matched their adult phantom counterpart to within ±0.1%. A similar technique was performed to rotate the legs of certain phantoms who had large thigh circumference targets.

For low total body mass phantoms, a limit was reached wherein the small target circumferences of the RST layer led to unavoidable intersections with the underlying internal organ meshes. For these phantoms, uniform two-dimensional scaling of all internal organs in the chest and abdomen, excluding the RST and skin layers, was performed in order to access small secondary parameters and overall total body mass targets. Vertices in the thoracic region were manually selected using a rectangular selection box and downscaled in discrete quantities (95% shrinkage to 75% shrinkage, in 5% increments). Initially, an abdominal rigging procedure similar to the one used for limb rotation was implemented, but this attempt ultimately failed since the automatic assignment of vertex weights is dependent upon object shape and therefore inconsistent among the abdominal organs.

### 2.3. Adult female breast modeling

**Figure 3.**
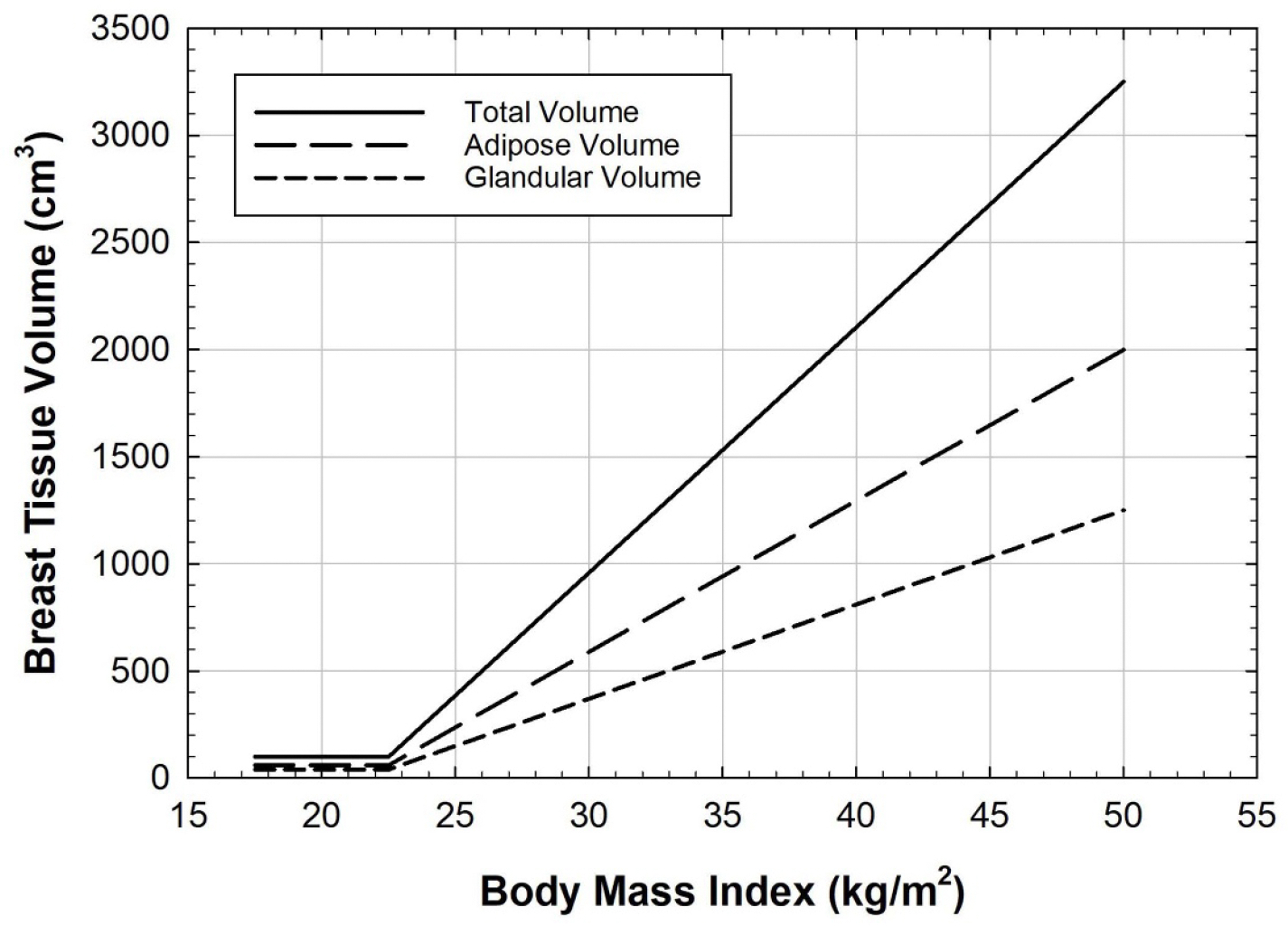
Breast volumes as a function of BMI.

Given the known correlation of breast volume with BMI in adult females (Coltman et al 2017), adipose and glandular tissues in the adult female phantoms were uniquely modeled based on each combination of standing height and total body mass. Once general modeling was completed for the 170 cm standing height series, as described in §2.2, target volumes for breast tissues were calculated as a function of phantom BMI using the data provided in Coltman et al (2017) while assuming a glandularity equal to that in the adult female MRCP (40% glandular and 60% adipose). For low BMI phantoms in which glandular volume was predicted to be negative, a floor value (equal to the smallest nonnegative value) was used, meaning several of the lightest phantoms for a given standing height series shared the same breast volume values. The relationship between total (average), adipose, and glandular breast volumes and BMI is shown in figure 3. Beginning with the 170 cm tall upscaled adult female MRCP and the phantom’s known BMI, approximately 20 unique breast models were created manually, using a combination of 1D, 2D, and 3D scaling, as well as direct manipulation of mesh vertices. Once the breast meshes were complete, they were brought as close to the chest wall as possible while correcting mesh intersections with lymph nodes and the RST. The outer skin layers were deformed to closely overlay the adipose breast tissue and thus avoiding significant RST shielding.

### 2.4. Arm positioning for CT simulations

CT examinations are commonly performed in either an ‘arms-up’ (AU) or ‘arms-down’ (AD) position. To enable CT imaging simulations to properly account for the effects of arm positioning, each MRCP in the AD position was converted to the AU position using the anthropometric rigging procedure described in §2.2. The structures internal to the RST layer were used as a starting point to generate AU models for each standing height represented in the UF/MSK library. RST and skin layers in the AD phantom for a specific standing height and total body mass combination were then paired with a resized rig and converted to the AU position, and then combined with the rescaled AU internal meshes. For example, to create the 180 cm tall, 100 kg adult male in the AU position, the internal meshes of the adult male MRCP in the AU position were upscaled to 180 standing height and then combined with rigged and modified RST and skin layers for the 180 cm tall, 100 kg adult male in the AD position. This methodology was applied across the library to generate a companion library of phantoms in the AU position with identical heights and body masses. For any given height/body mass phantom, tissue masses in the AD and AU versions were generally matched to within 0.1% difference. The only exception to this was the 50 *μ*m radiosensitive skin layer; the mass of this tissue in the AU phantoms was relatively higher due to the larger surface area of the layer in the AU configuration.

### 2.5. CT source term development and validation

CT dosimetry is a notable example of the use of this new computational phantom library. This simulation first requires the modeling of a CT imaging system. A CT source term was developed in the Monte Carlo simulation code particle and heavy ion transport code system (PHITS) version 3.24 using the user-defined source feature (Kofler 2021), providing a description of the radiation output of a system across different system settings. Four tube voltage options (80 kVp, 100 kVp, 120 kVp, 135 kVp), two bowtie filter options (medium, large), and three collimation options (10 mm, 20 mm, 40 mm) were developed virtually and validated with physical volume CT dose index (CTDI_vol_) measurements using a Canon Aquilion ONE/GENESIS CT system. Though this system does have an option for use of a small bowtie filter, to date this filter has not been used clinically at University of Florida Health and was therefore omitted from the source-term definition.

**Figure 4.**
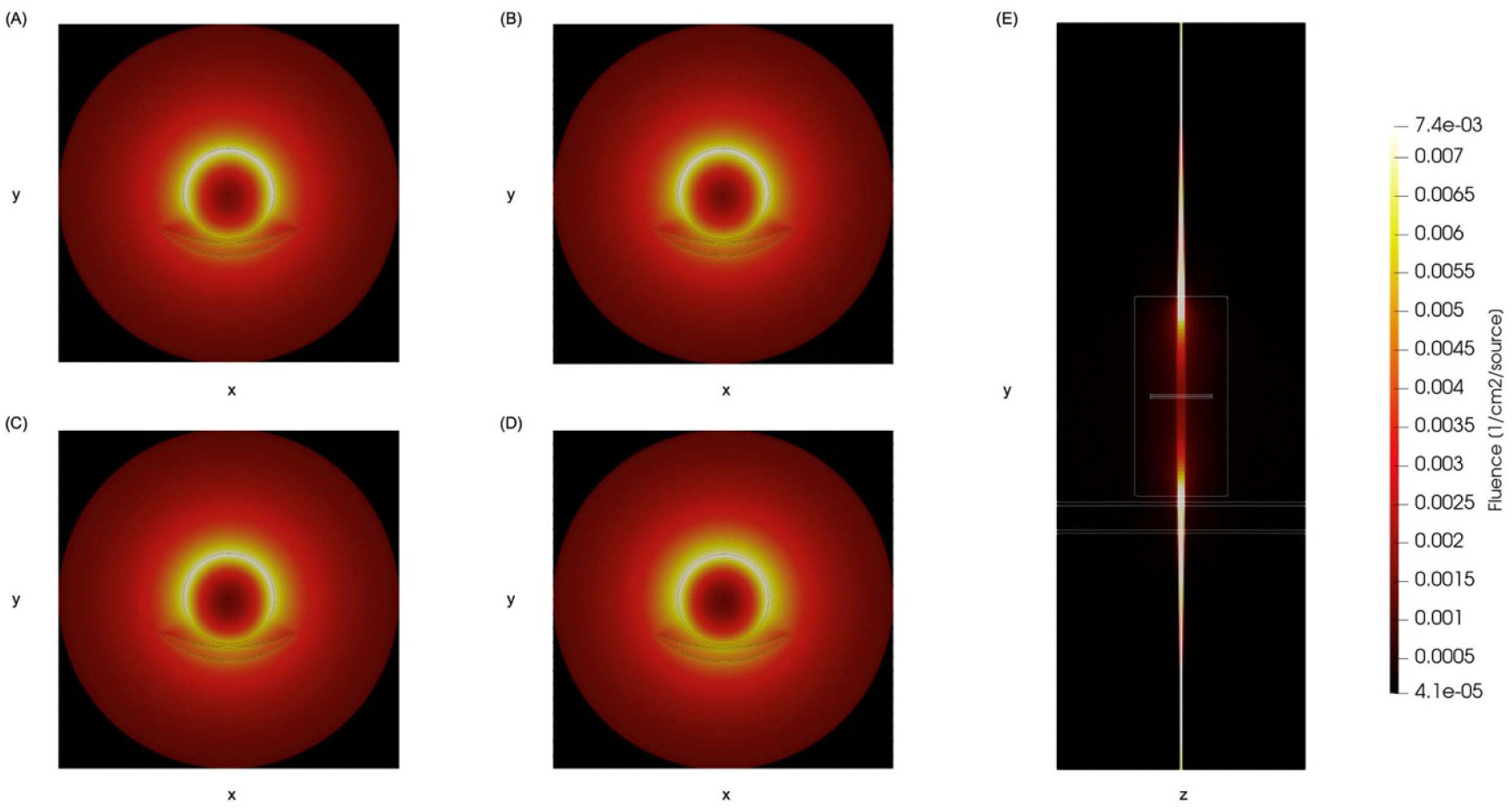
Example geometry output from PHITS showing a standard CT simulation (with 10 mm beam collimation) performed on a 32 cm CTDI phantom using the medium bowtie filter at (A) 100 kVp and (B) 120 kVp, and (C) the large bowtie filter at 100 kVp and (D) 120 kVp. In parts (A) through (D), the phantom, ion chamber, and patient table are outlined in black. Part (E) shows a lateral view of the medium filter/120 kVp simulation, with geometric components outlined in white. Fluence in all plots is in units of 1 cm^2^/source.

For each technique factor, five different physical measurements using the clinical scanner were performed. These included (1) first half-value layer (HVL) measurements for different spectra, (2) lateral dose profiles, (3) beam widths (with penumbra), (4) free-in-air kerma measurements at isocenter, and (5) CTDI_vol_ measurements (Stepusin et al 2017a). These physical measurements were then used to iteratively create the source model according to the methodology of Turner et al (2009) and using an initial x-ray spectra provided in Spektr v3.0 (Punnoose et al 2016). While this source term is specific to the Canon Aquilion ONE/GENESIS CT system, the same approach can be applied to generate analogous source terms for other scanner makes and models. Figure 4 displays examples of the CT geometry as visualized with PHITS used to perform the virtual CTDI_vol_ measurements.

Dose tallies in PHITS were calculated with units of ‘mGy/source’ (interpreted as mGy/photon). Normalization factors for each combination of tube voltage (kVp), filter size, and collimation width were calculated to convert organ doses from mGy/photon to mGy/mAs. Further details are found in Kofler (2021).

Finally, organ doses resulting from scans performed with tube current modulation (TCM) were also estimated. A first principles TCM algorithm for dose reconstruction was developed in which air kerma to an annulus of air (with a radius exceeding that of the CT photon ring source) is tallied for each *z*-axis slice of the phantom. For every slice, this quantity was then assumed to be inversely proportional to the overall attenuation (across both *x* and *y* dimensions) of the slice. The set of attenuation correction factors (ACFs) generated in this way, for a given phantom and technique-factor combination, can then be normalized by either the maximum or average mAs value of the set. Organ/tissue doses for each slice are then scaled by this normalized ACF to produce final dose values. This approach accounts for both longitudinal and angular TCM but does not account for explicit differences in TCM algorithms as implemented by different CT vendors (Stepusin et al 2017a, 2017b).

Experimental validation of the CT source term was accomplished via direct comparison of CTDI values—edge, central, and weighted values—obtained via a PHITS geometric primitive model of the CTDI phantom (both 16 cm and 32 cm diameter) and direct physical measurements at the UF Department of Radiology. Comparisons are made at all combinations of the technique factors to include three collimation settings, two filter sizes, and four tube voltage. The results are given in table 6, and indicate that the CT source term model successfully predicted measured values within ±5%, with the majority of technique combinations giving simulated values to within ±3% of measurements.

### 2.6. CT simulations and dosimetry

To prepare the UF/MSK phantoms for axial CT slice simulations, a geometric model of the CT table was created in polygon mesh format. The table was modeled as a carbon shell filled with air, and an in-house script written using Blender’s Python application programming interface was used to position the table such that it was 1 mm from the most posterior vertex of the phantom. For phantoms exceeding the default 180 cm table length (such as many in the AU position), the script also extended the table such that its length was equal to the axial length of the phantom.

Tetrahedralized phantom models (including the CT table) were then imported into the radiation transport code PHITS, and axial slice simulations were performed across the entire length of the phantom for specific technique-factor combinations. Details of the radiation transport simulations using the PHITS code system are provided in table 7. Regardless of the collimation setting (10, 20, or 40 cm), each axial rotation was acquired with a *z*-axis spacing of 1 cm, thus giving the ability to properly place the start and end of the CT scan ranges across the phantom with a spatial accuracy of 1 cm, accordingly. Organ doses in mGy/mAs for each slice were then stored in a database. The database of slice-by-slice organ doses was then utilized to estimate individual organ doses of clinically relevant axial and helical exams. Doses from axial exams were then calculated by summing individual slices over the scan range of a given protocol and multiplying by a user-specified mAs value to obtain the organ dose in units of mGy. Doses from helical scans were then calculated using the same methodology with additional normalization by pitch to account for the fact that the helical trajectory of the x-ray source was not explicitly modeled. The expression to calculate organ doses (in mGy) resulting from a given clinical scan is written as:

(1) $$D_{\text{Organ}}(mGy)=\sum_{i=Z_{\text{start}}}^{Z_{\text{end}}}D_{i}*A_{i}*mAs*\left(\frac{\;\text{scan}\;\text{length}\;}{\;\text{pitch}*\text{collimation}*X}\right),$$

where i is the slice number (where, by convention, slice zero is the bottom of the feet), $Z_{\text{start}}$ and $Z_{\text{end}}$ are the start and end indices for the selected clinical scan, $D_{i}$ is the normalized dose (in units of mGy/mAs), $A_{i}$ is the unitless ACF to account for the potential use of TCM, X is the number of slices in the scan range, and *scan length, pitch*, and *collimation* are defined as usual. The inclusion of scan length, collimation, and X in this equation are used to normalize each slice to account for slice overlap that occurs at the 20 and 40 mm collimation sizes while using a 10 mm slice spacing. In this manner, users of this pre-computed CT dose library can obtain absolute values of the virtual patient organ dose in units of mGy by simple multiplication by the examination-specific mAs value for fixed tube-current scans and by the effective mAs and an attenuation scaling factor for TCM scans.

### 2.7. PHITS CT simulations of phantom library subset

Slice-specific CT organ doses were computed for a subset of phantoms in the library. CT simulations using the source term described previously were performed for adult females of 170 cm standing height using a medium bowtie filter, 120 kVp tube voltage, and 40-mm collimation, for both AD and AU versions of the phantoms (40 phantoms in total). Total organ doses were calculated by summing these slice-specific values according to common examination protocols and scan ranges based on anatomical landmarks published by the University of Florida Department of Radiology Standard Names for Imaging Procedures (UF SNIPs)^10^.

## 3. Results

### 3.1. Height and body mass distributions for final adult library

**Figure 5.**
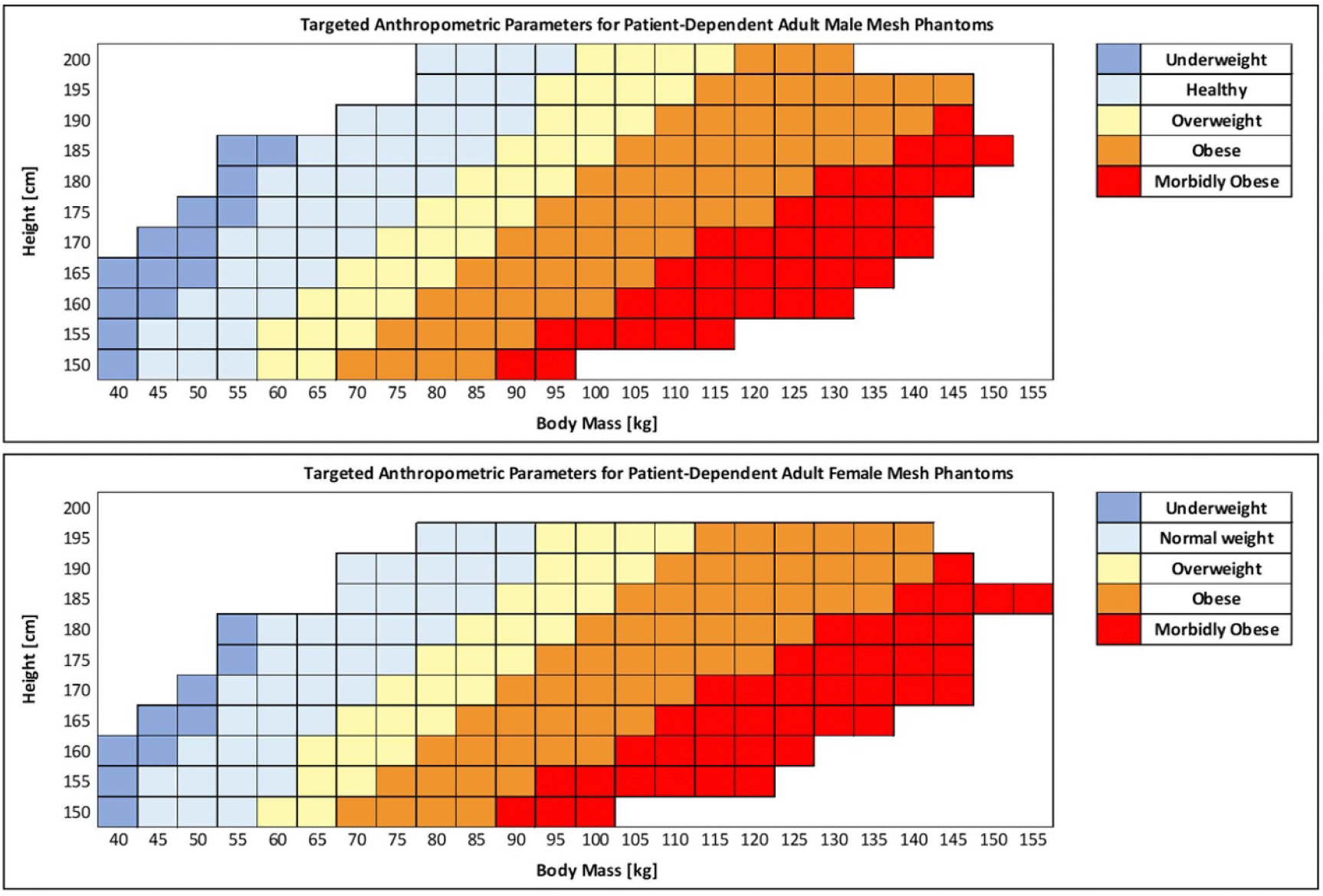
Final height and body mass distributions for adult males (top) and adult females (bottom). Blocks in the grids are color-coded based on BMI category.

The final height and body mass distributions for the adult library are shown in figure 5. There are a total of 186 adult male phantoms and 171 adult female phantoms. Standing and sitting heights were matched exactly to their respective targets, while total body masses were reached to within ±1%. A summary of the final masses for each tag region can be found in Annex C (males) and Annex D (females).

### 3.2. Polygon mesh and tetrahedral mesh phantom geometries

**Figure 6.**
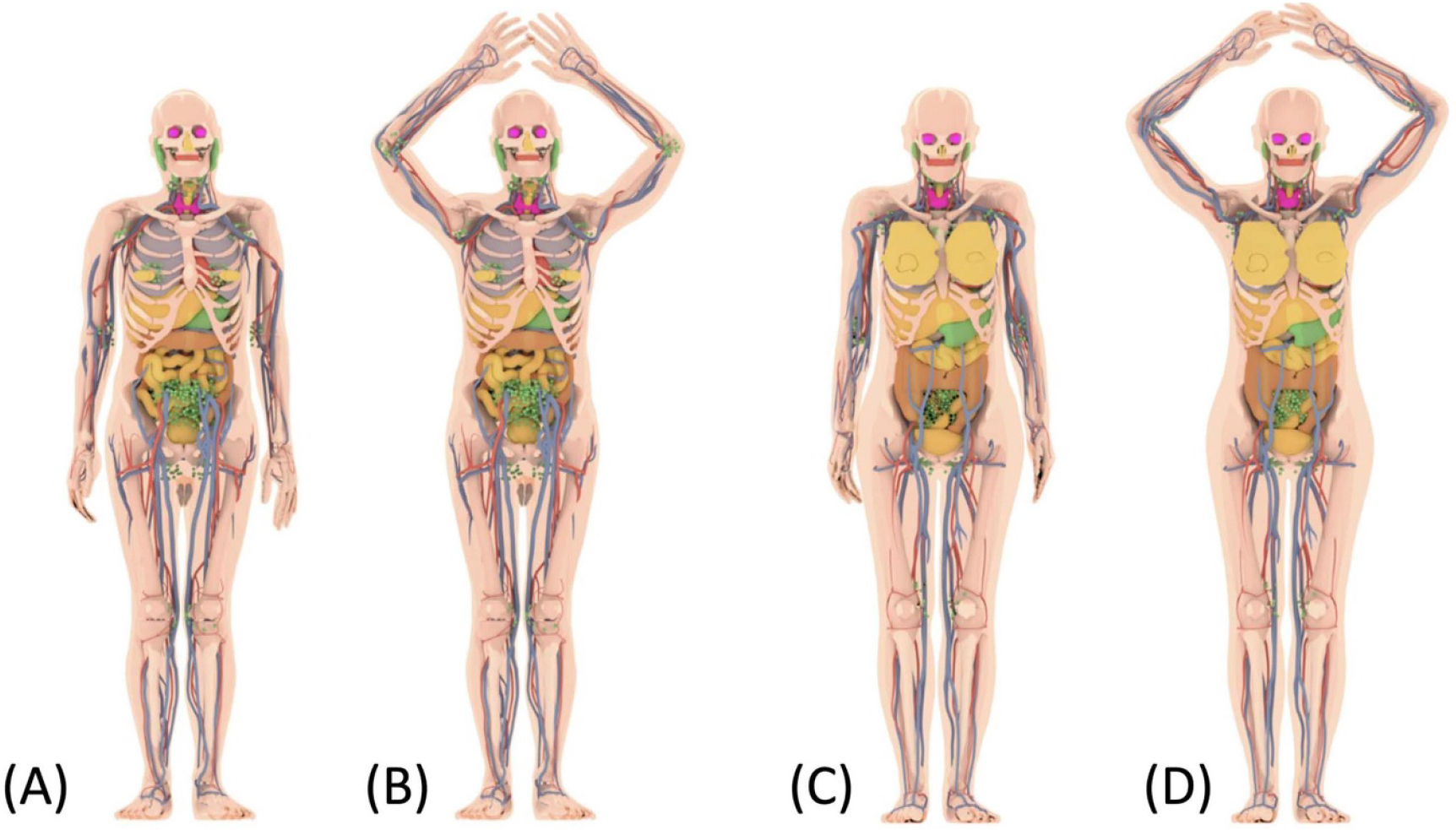
Polygon mesh adult MRCPs. (A) AD adult male, (B) AU adult male, (C) AD adult female, (D) AU adult female.

**Figure 7.**
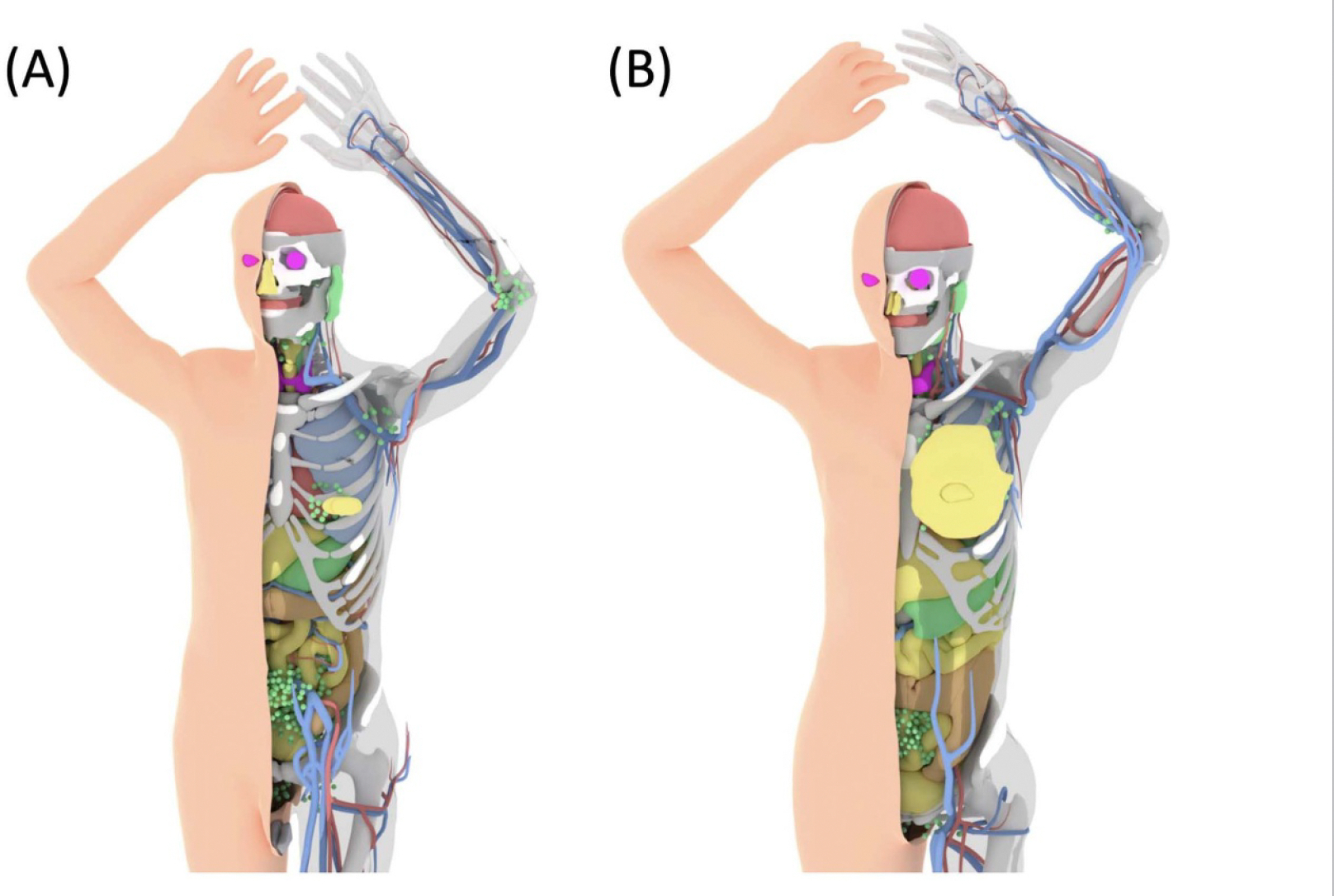
Polygon mesh adult MRCPs in the AU position. Shown are the adult male (A) and adult female (B).

**Figure 8.**
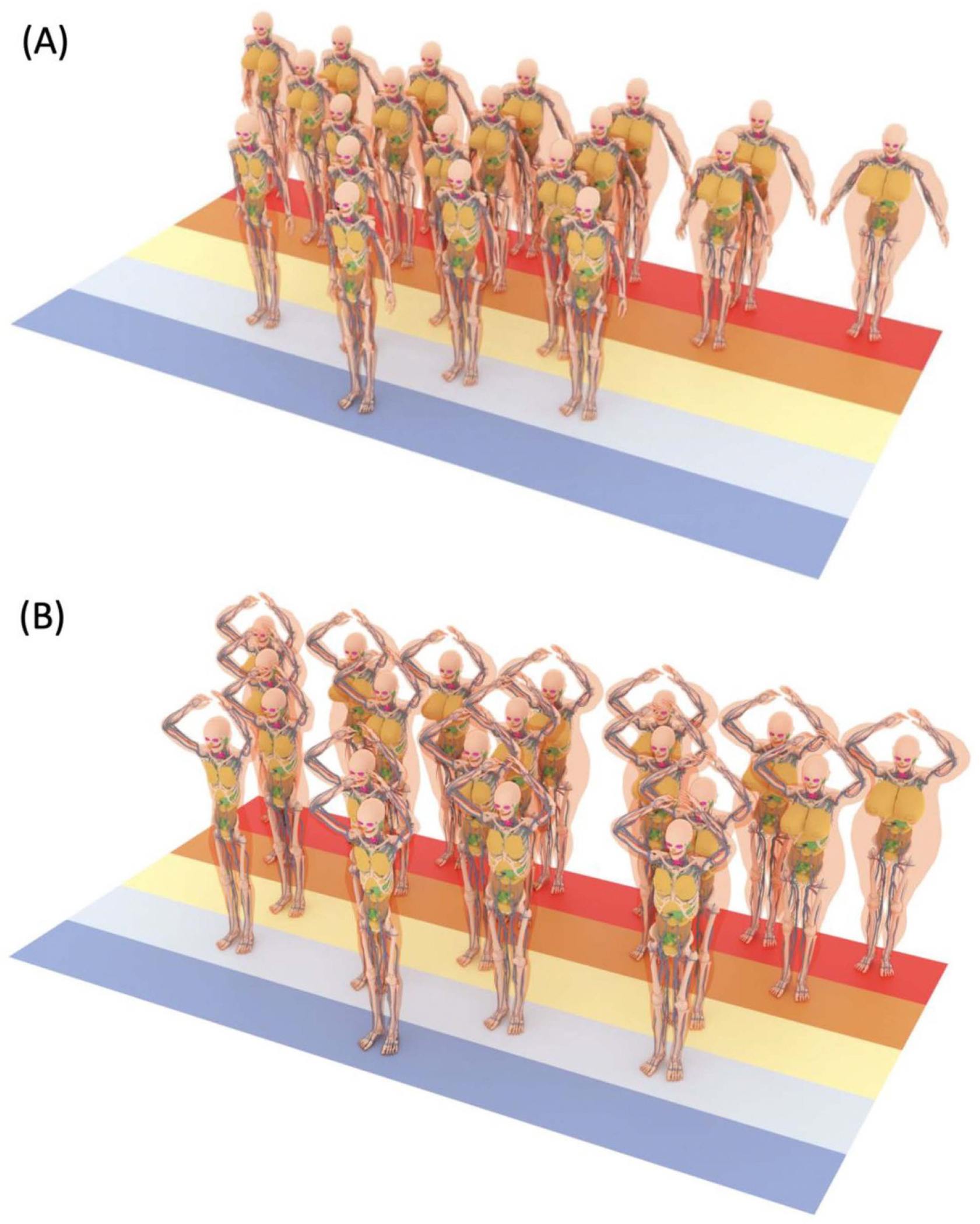
Visualizations of 170 cm standing height adult females in the (A) AD and (B) AU positions. Phantoms are sorted in rows based on BMI category.

**Figure 9.**
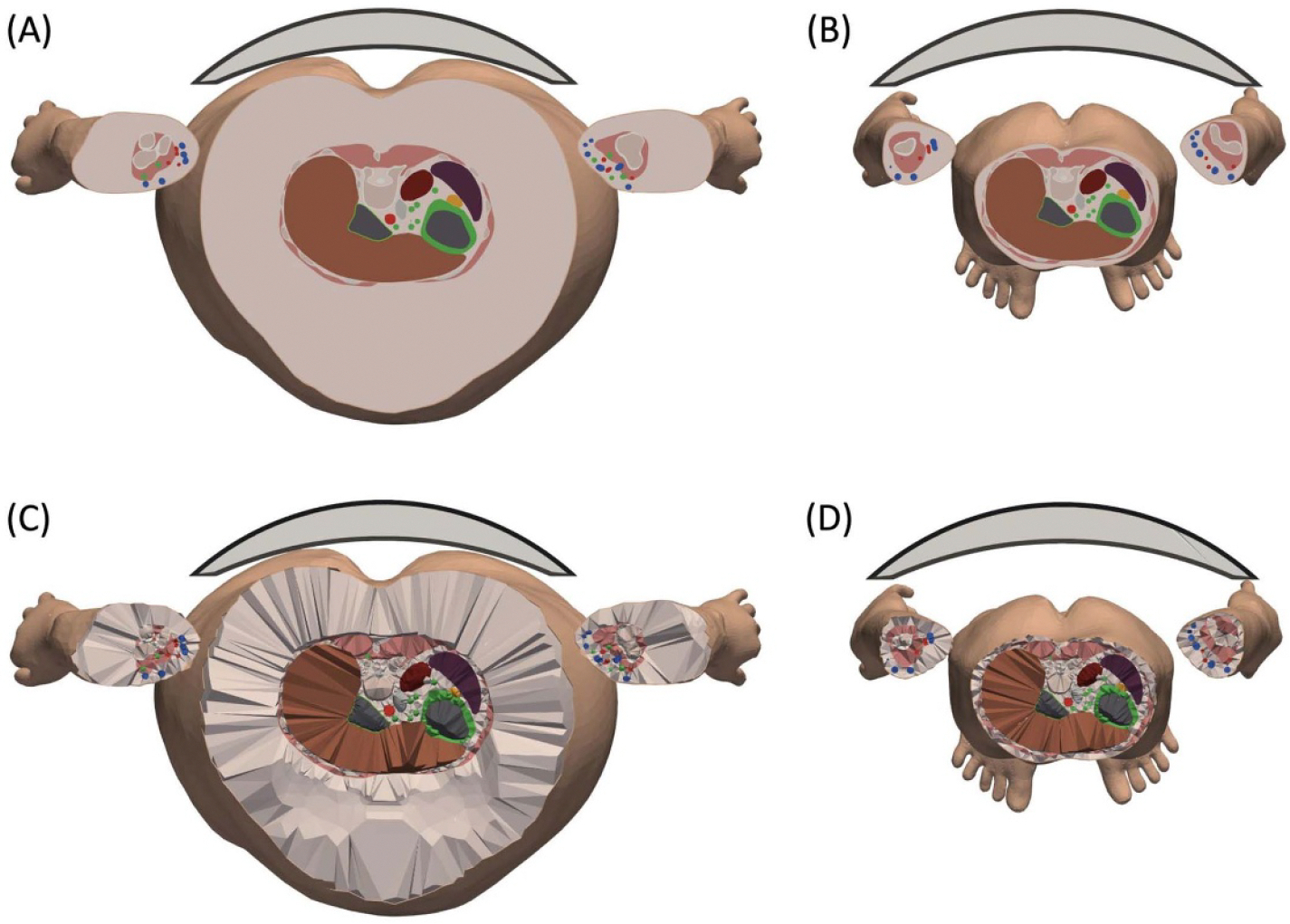
Axial views of tetrahedral mesh phantoms of 170 cm standing height adult females at (A) 145 kg (flat cut), (B) 50 kg (flat cut), (C) 145 kg (crinkle cut), (D) 50 kg (crinkle cut). Images generated in ParaView version 5.11.1.

**Figure 10.**
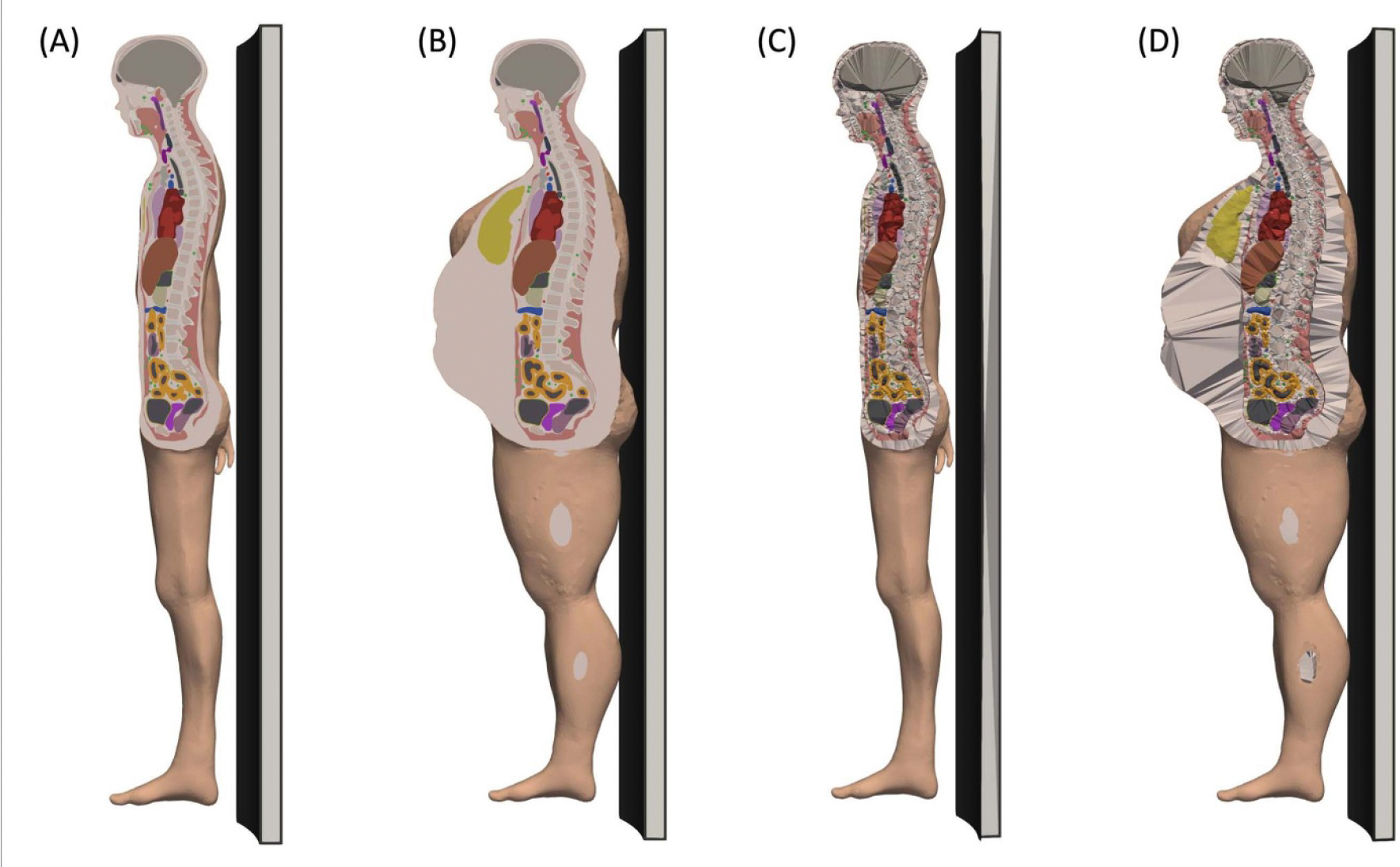
Sagittal views of tetrahedral mesh phantoms of 170 cm standing height adult females at (A) 50 kg (flat cut), (B) 145 kg (flat cut), (C) 50 kg (crinkle cut), (D) 145 kg (crinkle cut). Images generated in ParaView version 5.11.1.

Polygon mesh surface models are shown in figures 6 and 7 for the adult MRCPs in both the AD and AU positions. The subset of adult females used in CT simulations in this study are shown in figure 8 in rows that are color-coded based on BMI category. For this 170 cm standing height series of phantoms, the lightest (50 kg) and heaviest (145 kg) phantoms are shown in tetrahedral mesh format in figures 9 and 10.

### 3.3. CT dosimetry for phantom library subset

**Figure 11.**
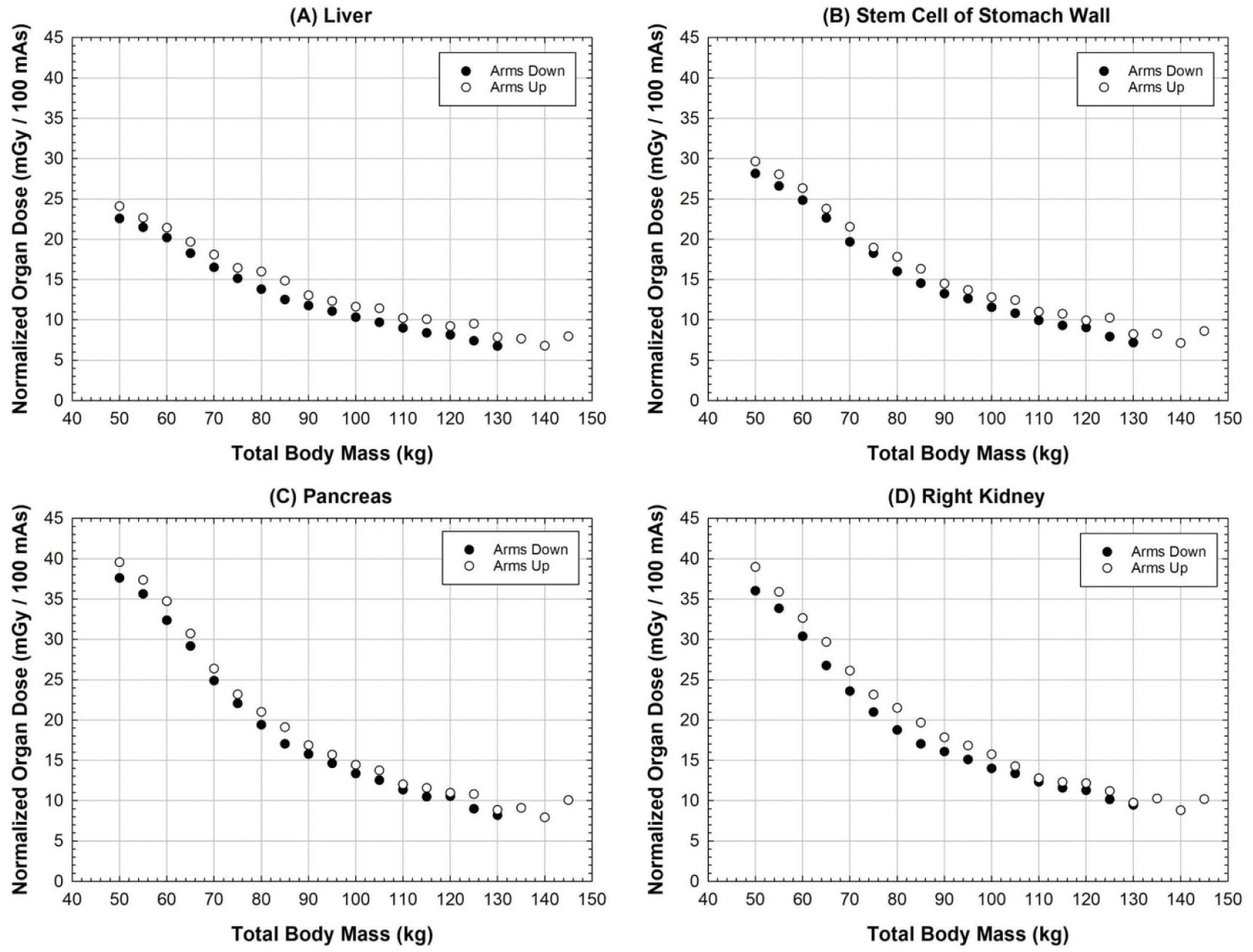
Normalized dose [mGy/100 mAs] as a function of total body mass resulting from an abdomen exam absorbed in (A) liver, (B) stem cells of stomach, (C) pancreas, and (D) kidney, for both AD and AU versions of the phantoms.

**Figure 12.**
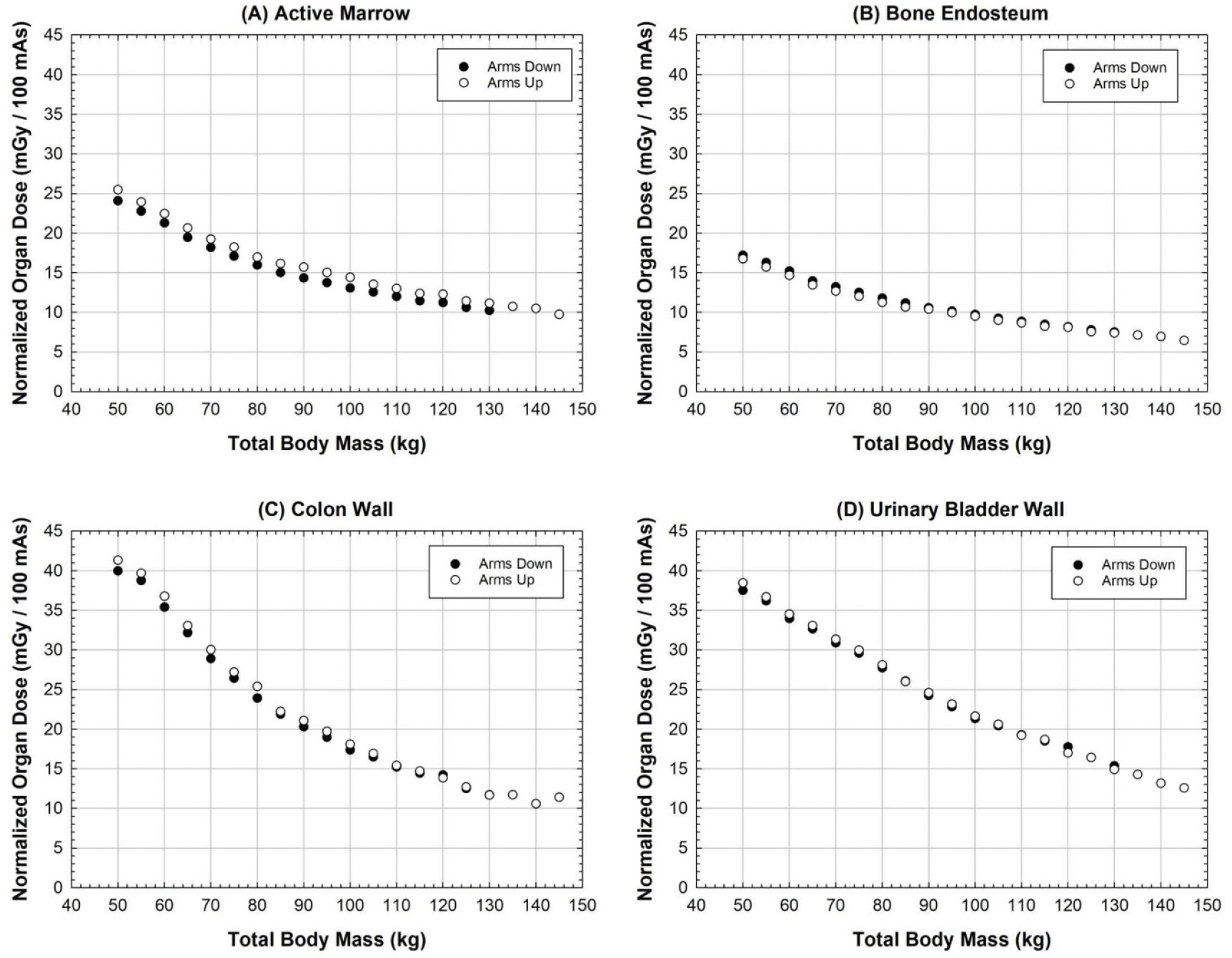
Normalized dose [mGy/100 mAs] as a function of total body mass resulting from a CAP exam absorbed in (A) red (active) marrow, (B) 50 mm endosteal region, (C) colon wall, and (D) urinary bladder wall, for both AD and AU versions of the phantoms.

**Figure 13.**
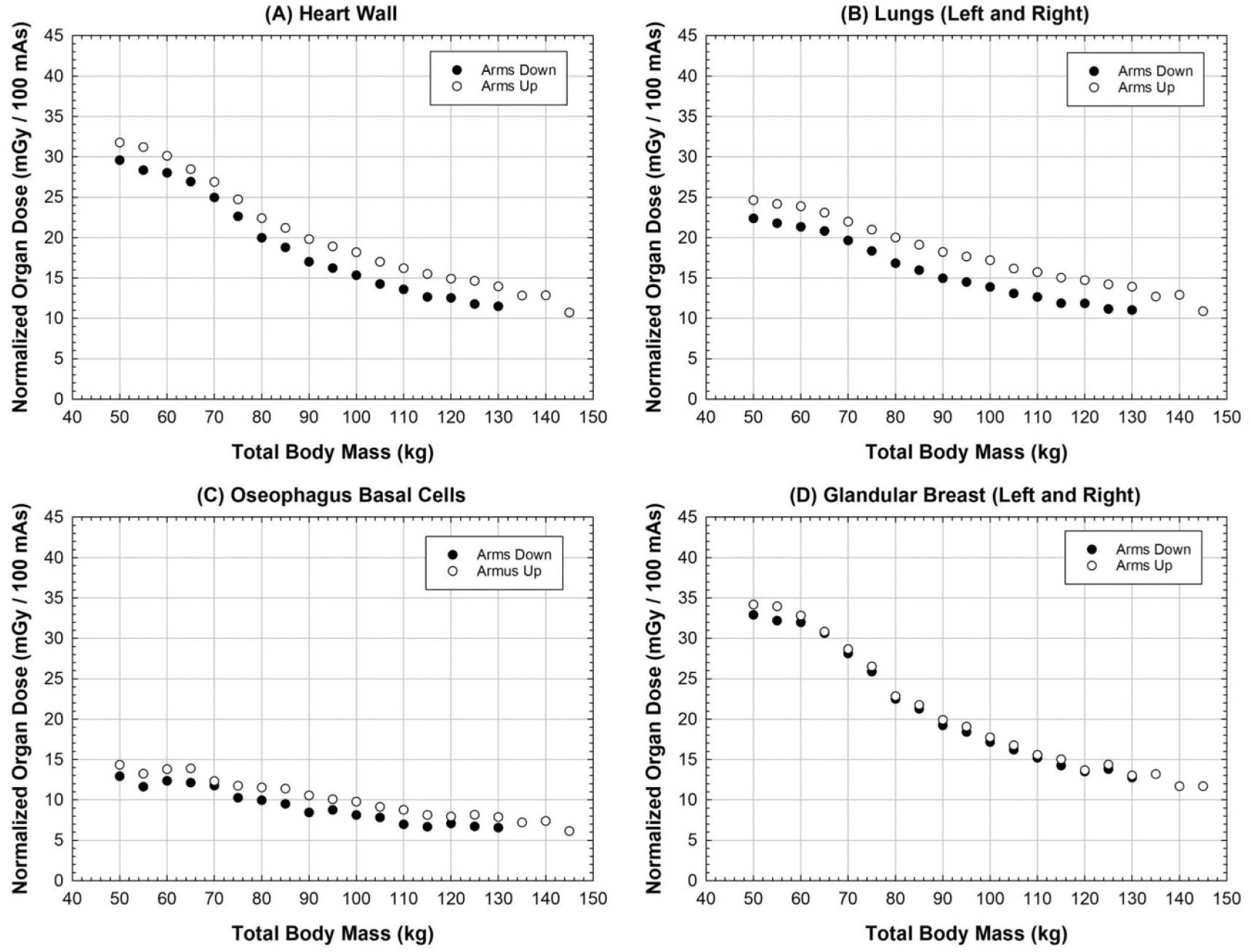
Normalized dose [mGy/100 mAs] as a function of total body mass resulting from a cardiac exam absorbed in (A) heart wall, (B) lung, (C) esophageal basal cells, and (D) glandular breast tissue, for both AD and AU versions of the phantoms.

**Figure 14.**
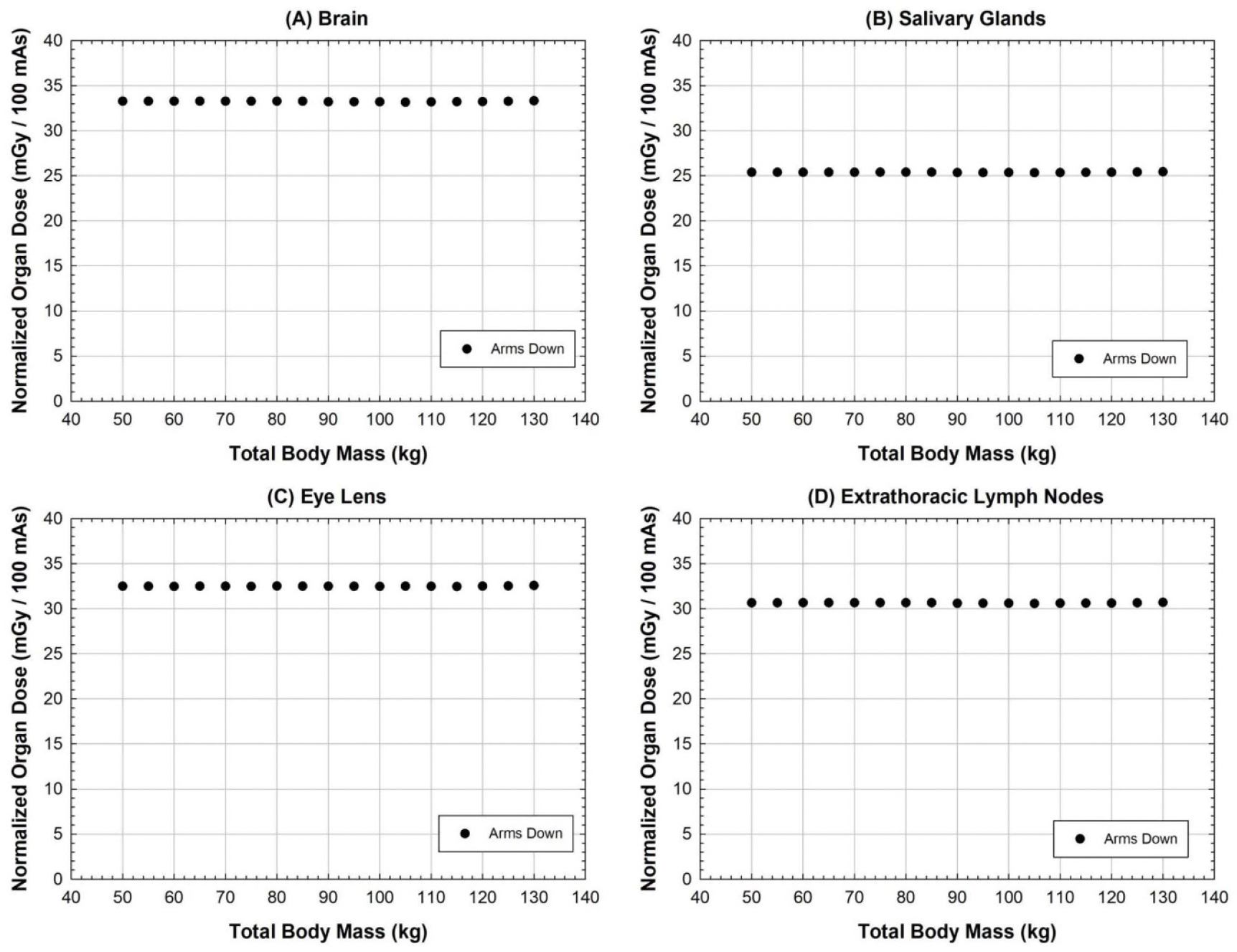
Normalized dose [mGy/100 mAs] as a function of total body mass resulting from a head/brain exam absorbed in (A) brain, (B) salivary glands, (C) entire lens of eye, and (D) extrathoracic lymph nodes, for AD versions of the phantoms (AU simulations not shown as head/brain exams are not performed in the AU position).

For each adult female in the CT simulation phantom subset, all organ doses in mGy, normalized to a fixed tube current-time product of 100 mAs, were computed for several common CT studies. Normalized doses to several major organs as a function of body mass resulting from abdomen, chest–abdomen–pelvis (CAP), cardiac, and head/brain exams are shown in figures 11–14, respectively. Simulations were performed in PHITS with 10^7^ particle histories per slice to achieve relative errors for in-field organ doses under 1%.

## 4. Discussion

### 4.1. Phantom, organ, and tag naming conventions

All phantom tag numbers and corresponding mesh object names for each organ and tissue are identical to those found in the MRCPs. Phantoms were named using the following convention: ‘UFMSK’, followed by the phantom’s subpopulation (‘AM’ or ‘AF’), standing height in centimeters, total body mass in kilograms, and arm position identifier. For example, an adult male from the UF/MSK library at 170 cm standing height and 100 kg total body mass in the AU position would be identified as the ‘UFMSK_AM_H170_W100_AU’ phantom.

### 4.2. Body morphometry parameterization, positioning, and BMI effects on CT organ doses

Compared to other existing and recently developed mesh phantom libraries, the UF/MSK phantom library is novel for several major reasons, including (1) coverage of heights, body masses, and body morphometries specific to the North American adult population, (2) a modeling approach driven by the assumption that gains in total body mass, for a fixed height, are mostly attributable to gains in SAT mass, and (3) extensive utilization of the novel deformability of mesh phantoms to produce models with realistically rotated limbs, including phantoms representing high-BMI individuals (requiring outward limb rotation to prevent intersection with the torso) a complete one-to-one companion library in the AU position. The assumption that, for a fixed height, gains in total body mass are mostly attributable to gains in SAT mass is justified: approximately 85% of all adipose tissue is SAT, while the remaining 15% is primarily visceral adipose tissue (VAT), and BMI is strongly correlated with SAT and much less so with VAT (Camhi et al 2011, Foster and Pagliassotti 2012, Pasanta et al 2021). Extreme changes to SAT mass and VAT mass in a single individual are not uncommon, but the latter is generally associated with metabolic or cardiovascular disorders (Shuster et al 2012). While gains in BMI can also be attributed to changes in muscle mass from physical training or certain pathologies, for example, the purpose of this phantom library is to represent population-averaged trends in body morphometry as parameterized by height and body mass (Sweatt et al 2024).

The CT organ doses computed for the subset of adult female phantoms examined in this study followed expected trends. In general, organ doses per 100 mAs decreased monotonically as a function of phantom BMI due to increasing adipose shielding. Phantoms in the AU position generally had higher organ doses than their AD counterparts due to the absence of arms that would otherwise attenuate the photon fluence incident on the torso.

### 4.3. Comparison of phantom library simulation subset to adult female MRCP

**Figure 15.**
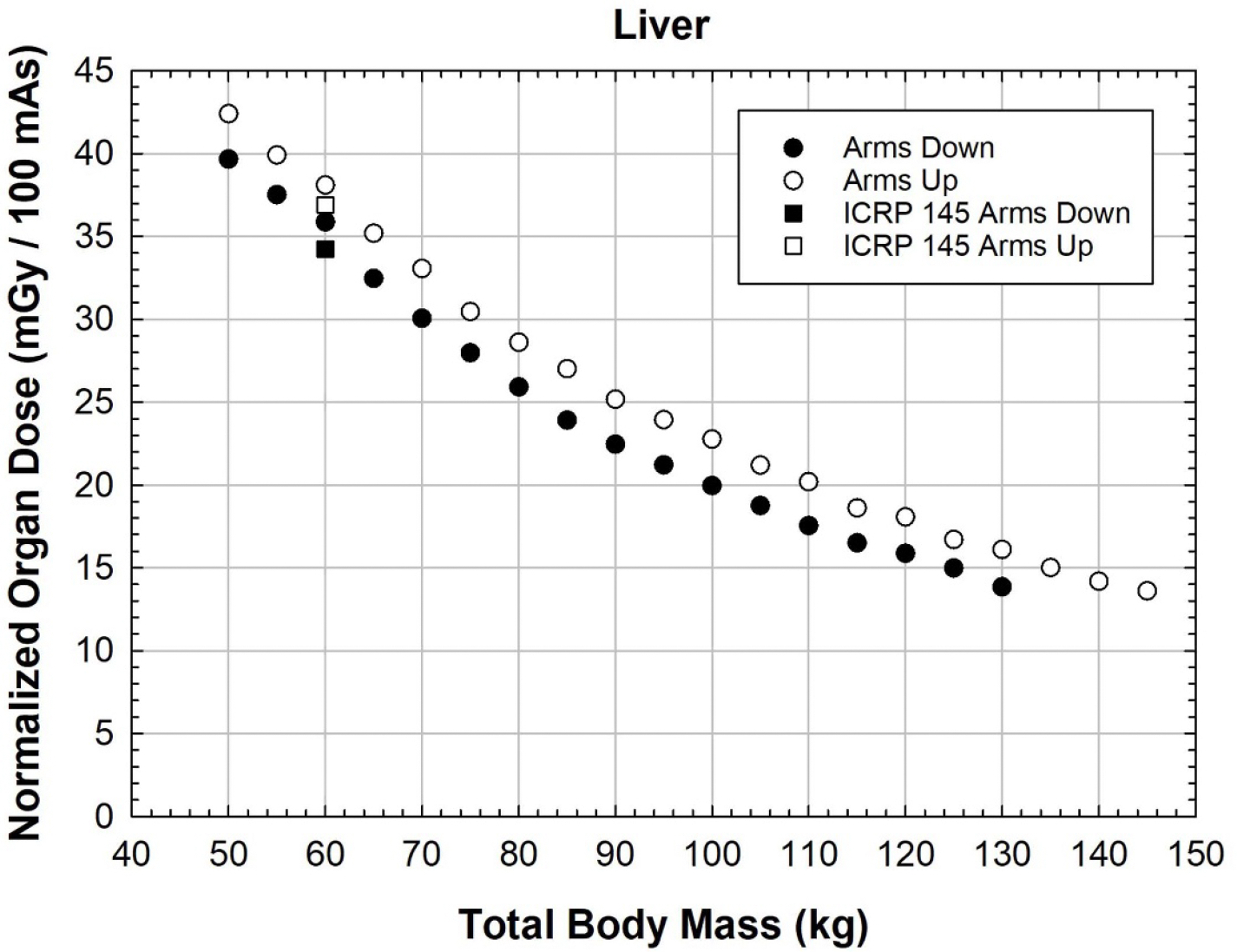
Normalized dose (mGy/100 mAs) to the liver as a function of total body mass resulting from a CAP exam, for both AD and AU versions of the phantoms (closed and open circles). Shown also (closed and open squares) are data for the AD and AU adult female ICRP Publication 145 MRCP.

In addition to the simulations performed on the subset of adult female phantoms from the library, similar simulations were performed on the ICRP reference adult female MRCP. Absorbed doses to the liver resulting from a CAP exam are shown in figure 15 for both the series of 170 cm tall UF/MSK adult females in their AD and AU postures (closed and open circles), as well as single values for the these two postures in the ICRP Publication 145 adult female MRCP (closed and open squares). For the CAP exam, the normalized absorbed dose to the liver in the ICRP 145 adult female was found to be 34.2 mGy/100 mAs in the AD posture and 36.9 mGy/100 mAs in the AU posture. While there is close agreement between the liver dose to the adult female MRCP and the liver dose to the 170 cm standing height, 60 kg body mass adult female (the adult female MRCP is 163 cm in standing height and 60 kg in total body mass), the liver dose to the MRCP differs significantly from those of other phantoms in the library. This illustrates the need for diverse phantom libraries and associated CT dose libraries as organ dosimetry on a single reference person is insufficient to assess dosimetry for specific patients whose body morphometries may deviate substantially from ICRP reference values. As shown in figure 15, normalized absorbed doses to the liver can range from a high of 39 and 43 mGy/100 mAs for the skinner phantoms (AD and AU, respectively) to a low of 14 and <16 mGy/100 mAs, in these same two arm positions for the more obese phantoms.

### 4.4. Limitations of the UF/MSK phantom library

A key assumption in the construction of the UF/MSK phantom library is that for a given sex at a fixed standing height, changes in total body mass were due primarily to changes in SAT as delineated by the RST layer. This means that a majority of phantoms in a given height series share the same lean body structures such that organ size, shape, and relative position are generally fixed and derived from uniform scaling of these structures in the associated MRCP. An exception to this is found in the lower-body mass phantoms of a given height series for which axial abdominal scaling had to be performed (see the flowchart of the modeling process in figure 1). Several studies have shown that the strongest determinant of thoracic organ cranio-caudal length (i.e. z-length in the phantoms) is body habitus length, not body mass or BMI (Emamian et al 1993, Chow et al 2016, Pasanta et al 2021). This, paired with evidence that changes in BMI for a fixed height are mostly attributable to changes in SAT mass, support this axial *x*–*y* scaling approach. For the subset of adult female phantoms with simulated CT doses shown in figure 15, liver masses ranged from 1.354 kg to 2.115 kg (see Annex D), with only the three lowest mass phantoms having liver masses less than the non-axially scaled mass (2.115 kg). Despite this apparently large range in organ mass, the trend in organ dose is smooth, continuous, and monotonic as a function of total body mass, supporting the claim that axial 2D scaling is the appropriate method for modeling extremely low-BMI individuals. Furthermore, while certain pathologies, such as fatty liver disease, are correlated with obesity and therefore BMI, the purpose of the UF/MSK phantom library is to establish a set of models representative of healthy adults and population-level trends in their outer body and organ morphometries. Thus, when matching a patient to a phantom from the library, only outer body shape and size can be reasonably approximated by the phantom, while internal organ anatomy provides some level of organ size, shape, and position uncertainty relative to the ‘real’ patient to be represented.

### 4.5. Limitations regarding AU positioning

When constructing the AU companion library, the rigging process resulted in twisting and compression of tissues being deformed including the skeletal muscle, RST, skin layers, major arteries, major veins, and lymph nodes. This effect was amplified around the shoulders, where the humeral head serves as an origin of rotation with many degrees of freedom, while distortions near simpler joints such as the elbow were minor and easily corrected. Though the masses of these organs were very closely matched (to within 0.1%) to those in the AD counterparts, the manual nature of this volume restoration led to minor inconsistencies among height series with respect to where mass was restored. For example, the RST surface mesh was manually expanded around the shoulders and back of each AU phantom to offset the torsional distortion of vertices, and the degree to which this was needed depended on the original input geometry and the geometry of nested meshes such as the muscle and vasculature. The dosimetric impact of these inconsistencies can be seen in the less smooth trends in organ doses with increase body mass of the AU plots in figures 11–14, which contrast with the smoother organ dose profiles of the AD phantoms.

### 4.6. Limitations in the CT simulations

Though not an issue with phantom geometry, CT simulations for certain AD phantoms could not be performed due to the size of the virtual CT bore. When importing tetrahedral geometry into PHITS, a ‘phantom box’ must be defined using the phantom’s bounding box (i.e. a rectangular prism with its extent defined by the phantom vertices’ *x, y*, and *z* maxima and minima). For high-mass AD phantoms, outward rotation of the arms led to phantom box geometries with bounds exceeding the radius of the photon ring source in our source term, precluding Monte Carlo simulation. Since the arms generally do not define the phantom box for the AU phantoms, CT simulations for all AU phantoms could be performed using our Monte Carlo CT source term.

### 4.7. Incorporation of dosimetry data into MIRDct and future studies

Slice-specific CT organ dose data generated using the UF/MSK phantoms from the library will be incorporated into a future version of the freely-available MIRDct software code—a CT dosimetry module that is part of a powerful open-access software suite found at the website MIRDsoft.org (Ramos et al 2022). This software is being developed in collaboration with Memorial Sloan Kettering Cancer Center and the Society of Nuclear Medicine and Molecular Imaging’s Committee on Medical Internal Radiation Dose (MIRD), and will be populated with Monte Carlo CT organ dosimetry data as they are generated.

While breast volume in the adult female phantoms in the UF/MSK library was parameterized by BMI, the breast glandularity (40%) was kept uniform among all phantoms and inherited from the adult female MRCP. However, mammographic studies show substantial inter-individual variability linked to age and BMI, among other factors, implying the need for further investigation of breast doses for varying adipose-to-glandular ratios (Checka et al 2012, Sprague et al 2014, Caballo et al 2022). Future research directions will include the establishment of adult female phantoms (both pregnant and non-pregnant, utilizing the forthcoming pregnant female MRCP series) with varying BMI, breast volume, and breast glandularity to assess the impact and interactions of these factors in the context of CT exposure (Shin et al 2025).

The method for Monte Carlo source term development (described in §2.5) is based on the one proposed by Turner et al (2009), and while generalizable to other scanners, can be complex and time-consuming. An alternative method for converting CT dosimetry estimates is more commonly used and involves simple scaling of organ dose by the ratio of CTDI_vol_ values from the current scanner on which the source term is based to the uncharacterized target scanner (Turner et al 2010). Thus, given an existing set of CT organ doses, one can convert them to the predicted values for a different scanner with knowledge only of that scanner’s CTDI_vol_ for a given protocol. Future efforts will include the establishment of a database of CTDI_vol_ values for most modern CT systems, with varying tube potentials, collimations, x-ray bowtie filters, and phantom sizes, from all major vendors. This database will be integrated in future releases of MIRDct to provide organ dose estimates for a user-selected scanner (or user-defined CTDI_vol_) using this renormalization approach.

Due to proprietary intellectual property concerns, vendors due not typically disclose the full quantitative or technical details of their TCM algorithms, making it necessary to perform some sort of physical measurement-based characterization of ACFs if one desires dose estimates which are as system-specific as possible. The current first principles TCM attenuation correction method, involving the calculation of ACFs, is based solely on Monte Carlo tallies of exiting air kerma for the Canon-based source term. To physically validate these estimates of ACFs, data describing tube current output and resulting measurements of dose to a physical phantom must be reconciled with coupled dose estimates from a digital twin of this phantom. Currently, an ongoing effort is the construction of a ‘TCM Challenge Phantom’ comprised of variably sized segments of high-density polyethylene with bores for optically stimulated luminescence dosimeters (OSLDs) at different depths from the phantom surface. Physical CT dose measurements using the OSLDs will be used to refine the ACFs on a scanner-specific basis.

The phantoms developed in the present study are generally referred to as *patient-dependent*. In medical dosimetry applications, this means that a patient being exposed to radiation is matched to the phantom which is most similar in terms of height and body mass (Geyer et al 2014). This phantom, representing the population-averaged individual for that demographic, is almost certainly superior to a single reference individual in terms of fidelity to the patient but still lacks absolute specificity with respect to the individual’s anatomy. To better model the body habitus of the patient and achieve more accurate dosimetry estimates, a reference or patient-dependent phantom can be morphed to match just the outer body contour of the subject (*patient-sculpted phantom*) or outer body contour and segmented organ contours (*patient-specific phantom*). Clearly, the ideal scenario is to construct a patient-specific phantom by deforming a patient-dependent phantom based on a whole-body tomographic image of the patient, though historically this has been impractical, if not impossible, due to the difficulty in deforming traditional phantom geometries and the manually intensive and time-consuming nature of organ segmentation (Carter et al 2021). However, given the recent advent of deep learning-based autosegmentation algorithms and fully mesh-based phantoms, it is now possible to construct patient-specific phantoms with minimal human intervention (Wasserthal et al 2023). In an ongoing project at the University of Florida, an automated workflow is being developed for patient-specific phantom generation using the UF/MSK phantoms, partial-body patient CT images, and the autosegmentation tool totalsegmentator. Autosegmented organ contours are used as morphing targets for deformable image registration with the corresponding organs in a height/body mass/sex-matched UF/MSK phantom, and the resulting displacement vector fields are applied to the nodes of the tetrahedral mesh such that the final phantom closely conforms to the patient’s external and internal anatomy (Dawson et al 2024).

## 5. Conclusion

The UF/MSK adult phantom library, the largest tetrahedral library of its kind, has been constructed with 357 mesh-based phantoms in each of the AD and AU positions. These patient-dependent phantoms have primary and secondary morphometric parameters representative of the North American population, covering the 5th through 95th percentiles of both standing height and total body mass, with additional phantoms at both height and body mass extremes based on prior clinical applications of the library’s predecessor (the UF/NCI phantom library). A Monte Carlo radiation transport source model was designed and physically validated prior to CT simulations on a subset of the phantoms from the adult female subpopulation. Organ doses calculated with this implementation followed predictable trends as a function body mass and arm positioning. A pre-computed, slice-specific CT dose library resulting from simulations on the full phantom library will be used to populate the open-access CT dosimetry software MIRDct to be added to the suite of medical dosimetry software codes freely available at MIRDsoft.org.

## Supplementary Material

Annex A

Annex B

Annex C

Annex D

Supplementary material for this article is available online

## Figures and Tables

**Table 1.** The anthropometric parameters to which ICRP mesh reference phantoms may be matched to populate the UF/MSK mesh phantom library. A check mark indicates that this parameter is present in a given NHANES entry, while the open circle indicates that this parameter is absent in the dataset. The data from the continuous NHANES is given in individual two-year durations.

Anthropomorphic parameters	NHANES III	1999–2000	2001–2002	2003–2004	2005–2006	2007–2008	2009–2010	2011–2012	2013–2014	2015–2016
Standing height	✓	✓	✓	✓	✓	✓	✓	✓	✓	✓
Sitting Height	✓	○	○	○	○	○	○	○	○	○
Body mass	✓	✓	✓	✓	✓	✓	✓	✓	✓	✓
Calf circumference	○	✓	✓	✓	✓	○	○	○	○	○
Thigh circumference	✓	✓	✓	✓	✓	○	○	○	○	○
Waist circumference	✓	✓	✓	✓	✓	✓	✓	✓	✓	✓
Buttocks circumference	✓	○	○	○	○	○	○	○	○	○
Arm circumference	✓	✓	✓	✓	✓	✓	✓	✓	✓	✓
Ave sagittal Ab Diam	○	○	○	○	○	○	○	✓	✓	✓

**Table 2.** Linear equation parameters of slope, y-intercept, and coefficient of determination for adult sub-populations from NHANES, fitting secondary parameters in terms of one or a function of both primary parameters.

Phantom Sub-population	Primary parameter (cm or kg/cm)	Secondary parameter (cm)	*Fitted slope*	Secondary parameter *Y-intercept*	*R^2^*

Adult males	Mass-to-standing-height ratio	Arm circumference	38.38	14.83	0.80
Adult males	Mass-to-standing-height ratio	Average sagittal abdominal diameter	−0.46	21.40	0.00
Adult males	Mass-to-standing-height ratio	Butt circumference	99.90	52.76	0.87
Adult males	Mass-to-standing-height ratio	Max calf circumference	34.49	21.83	0.72
Adult males	Standing height	Sitting height	0.42	16.78	0.61
Adult males	Mass-to-standing-height ratio	Thigh circumference	55.83	25.45	0.72
Adult males	Sitting height	Upper arm length by sitting height	0.24	15.65	0.19
Adult males	Standing height	Upper arm length by standing height	0.21	1.44	0.47
Adult males	Sitting height	Upper leg length by sitting height	0.32	13.22	0.13
Adult males	Standing height	Upper leg length by standing height	0.29	−9.37	0.42
Adult males	Mass-to-standing-height ratio	Waist circumference	135.93	33.19	0.84

Adult females	Mass-to-standing-height ratio	Arm circumference	42.98	12.10	0.85
Adult females	Mass-to-standing-height ratio	Average sagittal abdominal diameter	−0.53	21.40	0.00
Adult females	Mass-to-standing-height ratio	Butt circumference	117.34	51.54	0.89
Adult females	Mass-to-standing-height ratio	Max calf circumference	34.86	21.81	0.71
Adult females	Standing height	Sitting height	0.45	11.67	0.64
Adult females	Mass-to-standing-height ratio	Thigh circumference	63.00	23.89	0.77
Adult females	Sitting height	Upper arm length by sitting height	0.21	17.19	0.15
Adult females	Standing height	Upper arm length by standing height	0.20	3.12	0.39
Adult females	Sitting height	Upper leg length by sitting height	0.36	8.21	0.16
Adult females	Standing height	Upper leg length by standing height	0.31	−11.70	0.39
Adult females	Mass-to-standing-height ratio	Waist circumference	126.68	36.88	0.83

**Table 3.** NHANES 5th and 95th standing height percentiles for each sub-population along with rounded minimum and maximum phantom standing heights in the UF/MSK adult phantom library. Axial scaling targets standing heights and corresponding sitting heights in intervals of 5 cm of standing height within the tabulated ranges.

	Standing height at 5th and 95th percentiles (cm)		Standing height range (cm)	
Sub-population	5th	95th	Minimum	Maximum

Adult males	161.2	186.9	160	190
Adult females	148.5	172.2	150	175

**Table 4.** For each of the adult female standing height bins, the 5th and 95th percentile total body masses are rounded to evaluate the minimum and maximum total body mass range.

	Total body mass at 5th and 95th percentiles (kg)		Total body mass range (kg)	
Height bin (cm)	5th	95th	Minimum	Maximum

150	45.6	92.6	45.0	95.0
155	48.0	101.3	50.0	100.0
160	50.2	108.1	50.0	110.0
165	52.4	116.3	55.0	120.0
170	54.8	124.5	55.0	125.0
175	58.6	133.1	60.0	135.0

**Table 5.** For each of the adult male standing height bins, the 5th and 95th percentile total body masses are rounded to evaluate the minimum and maximum total body mass range.

	Total body mass at 5th and 95th percentiles (kg)		Total body mass range (kg)	
Height bin (cm)	5th	95th	Minimum	Maximum

160	51.1	91.3	50.0	90.0
165	55.3	99.3	55.0	100.0
170	59.8	107.5	60.0	110.0
175	62.5	116.8	60.0	120.0
180	65.7	128.7	65.0	130.0
185	70.0	138.2	70.0	140.0
190	74.2	145.6	75.0	145.0

**Table 6.** Relative percent differences of simulated and measured CTDI100,edge, CTDI100,center, and CTDIvol usingthe Canon Aquilion ONE/GENESIS CT source term in PHITS.

				Relative percent difference		
kVp	Filter	CTDI phantom (cm)	Collimation (mm)	CTDI_100,edge_	CTDI_100,center_	CTDI_W_

80	M	16	10	4.25	2.68	3.75
		32	10	4.05	−3.68	2.53

	M	16	20	1.31	0.56	1.08
		32	20	1.29	−2.27	0.59

	M	16	40	3.11	0.41	2.26
		32	40	5.17	−5.11	3.12

100	M	16	10	1.22	3.04	1.81
		32	10	1.85	−0.35	1.37

	M	16	20	−0.42	2.15	0.40
		32	20	−1.00	−0.64	−0.93

	M	16	40	−3.18	3.21	1.21
		32	40	−1.00	−1.05	−1.01

	L	16	10	−2.75	−1.04	−2.19
		32	10	−2.04	−4.47	−2.55

	L	16	20	−0.82	1.83	0.03
		32	20	−1.00	−0.84	−0.99

	L	16	40	−1.40	4.01	0.29
		32	40	−1.00	−1.05	−1.01

120	M	16	10	−0.73	−3.00	−1.46
		32	10	4.90	−4.84	2.69

	M	16	20	0.71	−2.11	−0.20
		32	20	4.84	−3.39	3.03

	M	16	40	3.02	−2.24	1.31
		32	40	1.56	−4.18	0.29

	L	16	10	0.07	−2.39	−0.74
		32	10	3.98	−4.27	2.21

	L	16	20	1.23	−3.11	−0.18
		32	20	−0.01	−1.95	−0.42

	L	16	40	1.80	−2.36	0.45
		32	40	3.89	−0.31	3.00

135	M	16	10	−0.31	2.21	0.51
		32	10	−3.14	−2.00	−2.89

	M	16	20	−0.53	3.66	0.81
		32	20	−1.21	0.49	−0.82

	M	16	40	−4.62	4.60	−1.74
		32	40	−1.90	−3.17	−2.19

	L	16	10	−3.26	0.46	−2.05
		32	10	−4.09	−2.06	−3.65

	L	16	20	1.34	2.93	1.86
		32	20	−0.53	−0.64	−0.53

	L	16	40	−3.70	1.83	−1.97
		32	40	−2.37	−2.67	−2.43

**Table 7.** Details of the transport computations and data post-processing in this study (Hirayama et al 2005, Johnson et al 2011, Sato et al 2018, Kofler 2021).

Item	Description	References
Code and version	PHITS v3.24	Sato et al (2018)
Source description	Custom source routine to sample particle energy and angle from the virtual x-ray source as described in Kofler 2021.	Sato et al (2018), Kofler (2021)
Cross sections	EGS5 for photons and secondary electrons	Hirayama et al (2005)
Transport parameters	Secondary electrons followed photon interactions with an energy cutoff of 1 keV	Sato et al (2018)
Variance reduction	No variance reduction techniques were utilized in this study.	
Statistical uncertainties and history numbers	Photon histories were set at 10^7^ histories which were shown to given absorbed dose and photon spongiosa fluences to within a relative error within 1% for those tissues within the primary fan beam at the specific axial location across the phantom.	Sato et al (2018)
Data and post-processing	Absorbed dose was tallied in all soft tissues and photon fluence was tallied within all skeletal spongiosa regions of the phantoms. Slice-specific values of organ absorbed dose (mGy/mAs) were computed as the product of the PHITS absorbed dose tally (mGy/photon) and a normalization factor (photons/mAs) which itself was computed as the ratio of measured free-in-air kerma (mGy/mAs) to the simulated free-in-air kerma (mGy/photon). Active marrow and bone endosteum absorbed doses were computed using dose response functions from Johnson *et al* (as modified by ICRP Task Group 113) for incident photons.	Sato et al (2018), Johnson et al (2011)

## Data Availability

The data of the study will be made available upon request of the authors. PHITS radiation transport files for all Monte Carlo radiation transport simulations conducted herein will be made available at a publicly accessible GitHub repository. The computational mesh phantoms from the UF/MSK adult phantom library will be made publicly available in the near future at MIRDsoft.org. All data that support the findings of this study are included within the article (and any supplementary information files).

## Footnotes

8 www.cdc.gov/nchs/nhanes.htm.

9 According to ICRP Publication 145, RST defines regions of adipose tissue and other miscellaneous tissues such as connective tissues.

10 https://protocols.xray.ufl.edu/live_protocols/snips/.
